# Supplementary material for: Exploring the outcomes of research engagement using the observation method in an online setting
Source: BMJ Open. 2023 Nov 21;13(11):e073953. doi: 10.1136/bmjopen-2023-073953 (PMC10668270; doi:10.1136/bmjopen-2023-073953)
Supplement: Supplementary data [file bmjopen-2023-073953supp001.pdf]

Supplementary Table 1

Observation Guide Descriptive field notes for every project group activity

1) Meeting No:

2) Name of the Observer:

3) Group Observing:

4) Date of Observation:

5) Time of Observation: From………. To……………. Total meeting running time:

6) Meeting platform:

7) Number of group members:

8) Names of the group members: (Use ID numbers)

9) Time when each member joins and exists - (capturing how long each stays)

10) Group member roles in each activity during the different research stages and the interaction including the direction of communication, frequency of interaction of each group member, who took the decisions, etc.

- How does the meeting start? What's the mood in the room? Is it all business or do members chat with each other before or after meetings? Does the meeting start on time? Do all members seem to understand the purpose of the meeting, why they are there, and the agenda for the day? Are PRPs consulted when scheduling the activities?
- Does everyone speak or just some group members? Look for familiarity in the conversation for e.g. chatting about previous work together.
- Who is taking all the decisions during the discussion? At what stages/decisions are PRPs being consulted? Did PRPs propose any changes? Capture the changes proposed (changes in study design, methods, materials, etc.) and if not followed, the reasons why if

possible. Do the PRP have any influence on the final decision? Are group members supportive of other opinions in the group? Are there any instances of appreciation of work, giving credit openly during the discussions? Note down examples of members showing respect and supportive of the different viewpoints. Do PRPs initiate and take part in the discussions? How are the non-attendees informed of the decisions? If their input included in the final decision?

- Do PRPs lead any of the discussions? Did you observe silent moments in the conversation? Note: silences do not mean that the group member is uninterested or dis-engaged. It might mean reflective thinking. Jot down your thoughts, and rationale if you make this observation and how the lead/others involve the member and keep the conversation moving.
- Does anybody in the group use terms not understood by the rest of the group? Are there any written materials distributed prior to the meeting for review and questions? If yes, what were these materials? Document any discussions about the written materials for e.g. what does this medical word mean? What is the reading level of these documents?
- What training/support is provided to the PRPs and researchers in the group to help them contribute to the research? Are training requirements discussed? Is there any support request made to the main study team? Is the support provided and how?
- Do any conflicts emerge? What are they? How does the group respond and address the conflict? Note down any challenges that group members faced while engaging on this project.
- What does the morale of the group seem to be at the end of the meetings? Are "next steps" discussed? Are next steps understood? Does anybody on the group linger after the

meeting has closed to talk or do they leave immediately? What kinds of comments are stakeholders making as they leave?

Specific points to capture related to the launch phase (getting to know each other, working together and sharing experiences to help the group understand the supports PRP need)

- What strategy is employed by the group to get to know each? Does any member know another in their group prior to the first meeting? Does the study Lead introduce everyone or do the group members introduce themselves? Or Both?
- Does the group have a formal/informal plan to work together including a communication strategy, roles and responsibilities on the project?? Who developed this plan? Are PRPs involved in its development? If there is no plan, how did the group members operate?
- Did the PRPs in the group share their experience about living with IBD?

Specific points to capture related to the design phase (refining the research question, designing the study, conducting the literature review, developing the study material):

- Do all group members understand the research question, purpose of the study? What changes have been made to the research question? How did the group operationalize the research question? Are PRPs involved in influencing this decision? What is the result of any changes made?
- Who is involved in designing the research? What is each group members contributions to the study design? Are any changes proposed to the study design? Are all suggestions incorporated into the design? Are group members in agreement with the final decision/ validate the changes? Capture the changes made to design based on PRP input and the reason for changes not made. Are group members flexible in making changes keeping the

rigor of the study in mind? Is everyone on board with respect to the final design and approach?

- Is there any mention about the design being straightforward, and easy to accomplish within the time available? Is there any discussion and concern about the study goals and methods? For e.g., need for additional resources like time, expertise etc. to make the study happen. How are these addressed?
- Does the qualitative study design, data collection and analysis accommodate and show respect for participant diversity? Describe the study participants from the conversations.
- Does the group have discussions regarding data/privacy protection?
- What is the role of PRPs in reviewing the literature?
- Who developed the study material? What is the PRP input in their development? Document the materials developed/reviewed by PRP, changes made and reason for those not made. Are the materials written in lay audience language? Evaluate the reading level of the materials.
- Document discussions of personal benefit and benefit to the IBD larger community (for e.g. gained deeper understanding of biologics, gained skills/new knowledge through this engagement, contribute towards the advancement of POR)

Specific points to capture during the Implementation phase (recruitment, data collection, analysis)

- Who is involved in the recruitment of study participants? Who obtained consent/ screened the participants? What is the PRP input during this phase?
- Who is involved in data collection (patients, researchers or a combination of both)?

- Do the recruited study participants represent the living or lived experiences of actual patients, including groups that are typically under-represented in research?
- Who is involved in the analysis of the data? How are PRPs involved in the analysis?
- Is data collection transparent? Does the group use verifiable methods in performing the research and in reporting the results? Does the group pay attention to the ethical rules, regulations, guidelines while conducting the study? (for e.g., informed consent process, patient-friendly materials, data confidentiality and privacy etc.)
- How are the analysis and results shared with the group? Are the results shared with group members in a timely manner? Are the results validated by the PRP? Did they voice any concerns with the results and how were these addressed? Is the study completed within the stipulated time?

Specific points to capture during the Knowledge Translation (KT) Phase (explaining or applying results to real world setting, sharing study findings)

- Are there any discussions or plans about KT? What is the PRP's input during this phase? Are the PRPs in agreement with the plans?

Reflective field notes for every group engagement activity

Document the following elements intended to contextualize what you have observed based on your perspective.

- Note ideas, impressions, thoughts about what you observed. Include insights about why you believe specific phenomenon occurred.
- Include any unanswered questions or concerns that you think are important to record for future observations.

- Record anything that comes to mind that has not been captured elsewhere.
