# Supplementary material for: Exploring the outcomes of research engagement using the observation method in an online setting
Source: BMJ Open. 2023 Nov 21;13(11):e073953. doi: 10.1136/bmjopen-2023-073953 (PMC10668270; doi:10.1136/bmjopen-2023-073953)
Supplement: Author's manuscript [file bmjopen-2023-073953.draft_revisions.pdf]

BMJ Open is committed to open peer review. As part of this commitment we make the peer review history of every article we publish publicly available.

When an article is published we post the peer reviewers' comments and the authors' responses online. We also post the versions of the paper that were used during peer review. These are the versions that the peer review comments apply to.

The versions of the paper that follow are the versions that were submitted during the peer review process. They are not the versions of record or the final published versions. They should not be cited or distributed as the published version of this manuscript.

BMJ Open is an open access journal and the full, final, typeset and author-corrected version of record of the manuscript is available on our site with no access controls, subscription charges or pay-per-view fees (http://bmjopen.bmj.com).

If you have any questions on BMJ Open's open peer review process please email info.bmjopen@bmj.com

BMJ Open

Critical outcomes of research engagement using the observation method

Journal:	*BMJ Open*
Manuscript ID	bmjopen-2023-073953
Article Type:	Original research
Date Submitted by the Author:	23-Mar-2023
Complete List of Authors:	Marshall, Deborah; University of Calgary Department of Community Health Sciences; IMAGINE SPOR Chronic Disease Network<br>Suryaprakash, Nitya; The University of British Columbia School of Population and Public Health<br>Lavallee, Danielle; The University of British Columbia School of Population and Public Health; Michael Smith Health Research<br>Barker, Karis; University of Calgary Department of Community Health Sciences<br>Mackean, Gail; University of Calgary Department of Community Health Sciences<br>Zelinsky, Sandra; University of Calgary Department of Community Health Sciences; IMAGINE SPOR Chronic Disease Network<br>McCarron, Tamara; University of Calgary Department of Community Health Sciences<br>Santana, Maria-Jose; University of Calgary and Alberta Health Services Department of Paediatrics; Alberta SPOR SUPPORT Unit<br>Moayyedi, Paul; McMaster University Faculty of Health Sciences; IMAGINE SPOR Chronic Disease Network<br>Bryan, Stirling; The University of British Columbia School of Population and Public Health; British Columbia SPOR SUPPORT Unit
Keywords:	Patient Participation, QUALITATIVE RESEARCH, HEALTH ECONOMICS, GASTROENTEROLOGY, Adult gastroenterology < GASTROENTEROLOGY, Inflammatory bowel disease < GASTROENTEROLOGY

SCHOLARONE™
Manuscripts

I, the Submitting Author has the right to grant and does grant on behalf of all authors of the Work (as defined in the below author licence), an exclusive licence and/or a non-exclusive licence for contributions from authors who are: i) UK Crown employees; ii) where BMJ has agreed a CC-BY licence shall apply, and/or iii) in accordance with the terms applicable for US Federal Government officers or employees acting as part of their official duties; on a worldwide, perpetual, irrevocable, royalty-free basis to BMJ Publishing Group Ltd ("BMJ") its licensees and where the relevant Journal is co-owned by BMJ to the co-owners of the Journal, to publish the Work in this journal and any other BMJ products and to exploit all rights, as set out in our licence.

The Submitting Author accepts and understands that any supply made under these terms is made by BMJ to the Submitting Author unless you are acting as an employee on behalf of your employer or a postgraduate student of an affiliated institution which is paying any applicable article publishing charge ("APC") for Open Access articles. Where the Submitting Author wishes to make the Work available on an Open Access basis (and intends to pay the relevant APC), the terms of reuse of such Open Access shall be governed by a Creative Commons licence – details of these licences and which Creative Commons licence will apply to this Work are set out in our licence referred to above.

Other than as permitted in any relevant BMJ Author's Self Archiving Policies, I confirm this Work has not been accepted for publication elsewhere, is not being considered for publication elsewhere and does not duplicate material already published. I confirm all authors consent to publication of this Work and authorise the granting of this licence.

For peer review only

Critical outcomes of research engagement using the observation method

Deborah A. Marshall,[1] Nitya Suryaprakash,[2] Danielle C. Lavallee,[3] Karis Barker,[1] Gail MacKean, [1] Sandra Zelinsky,[5] Tamara L. McCarron, [1] Maria J. Santana,[1,4] Paul Moayeddi,[6-7] and Stirling Bryan[2,3]

Affiliation:

1. Department of Community Health Sciences, University of Calgary, Calgary, Alberta, Canada
2. School of Population and Public Health, University of British Columbia, BC, Canada
3. Michael Smith Health Research, British Columbia, Vancouver, BC, Canada
4. Departments of Pediatrics, University of Calgary, Calgary, Alberta, Canada
5. Alberta, Strategy for Patient Oriented Research (SPOR) Support Unit, Alberta, Canada
6. IMAGINE SPOR Chronic Disease Network, Hamilton, Ontario, Canada.
7. Faculty of Health Sciences, McMaster University, Hamilton, Ontario, Canada.

Corresponding author(s):
Deborah A. Marshall, Cumming School of Medicine, University of Calgary, 3280 Hospital Drive NW, Health Research Innovation Centre (HRIC) Building, Room 3C58, Calgary, AB, T2N 4Z6, Canada. Email: damarsha@ucalgary.ca

ORCID IDs

Deborah A Marshall: https://orcid.org/0000-0002-8467-8008

Nitya Suryaprakash: https://orcid.org/0000-0001-8032-9129

Danielle C. Lavallee: https://orcid.org/0000-0002-5555-9675

Karis Barker: https://orcid.org/0000-0002-3530-566X

Gail MacKean: https://orcid.org/0000-0002-0209-4667

Sandra Zelinsky: https://orcid.org/0000-0001-5531-7660

Tamara L. McCarron: https://orcid.org/ 0000-0001-7242-1910

Maria J Santana: https://orcid.org/0000-0002-0202-5952

Stirling Bryan: https://orcid.org/0000-0001-7093-3058

For peer review only

Abstract

Objective: Explore the outcomes of research engagement (PE) in the context of qualitative research.

Design: Observation of engagement in two groups comprised of patients, clinicians and researchers tasked with conducting a qualitative preference exploration project. One group was led by a patient research partner (PLG) and the other by an academic researcher (RLG). A semi-structured guide and a set of critical outcomes of research engagement was used as a framework to ground our analysis.

Setting: Online.

Participants: Patient research partners (n=5), researchers (n=5), and clinicians (n=4), in total

Main outcome measures: descriptive and reflective observation data of engagement during meetings and email correspondence between group members.

Results: Both projects were patient-centered, collaborative, meaningful, rigorous, adaptable, ethical, legitimate, understandable, feasible, timely and sustainable. Patient research partners (PRPs) in both groups wore dual hats as patients and researchers and influenced project decisions wearing both hats. The PRPs lived experience in the RLG influenced many decisions than their counterparts in the PLG. They spent more time sharing their experience with biologics and helping their group identify a meaningful project question.

Collaboration seemed easier in the PLG than in the RLG. The PLG lead delegated tasks to stakeholders in the group ensuring that the patient voice was embedded in all aspects of the

For peer review only - http://bmjopen.bmj.com/site/about/guidelines.xhtml

research. Stakeholders volunteered to roles in the RLG. A formal literature review informed the design, study materials and analysis in the RLG while the formal review informed the study materials and analysis in the PLG. A PRP in the RLG and the PLG lead leveraged personal connections to facilitate recruitment. The outcomes of both groups were meaningful to all members of the group.

Conclusions: Engagement results in positive outcomes on the research in the context of qualitative research.

Strengths and limitations of this study

- We used direct observation of research engagement, which provided a more robust understanding of PRP roles and influence on the research.
- Observation was in an online environment, and overt (Group members were aware they were being directly observed in all project communications).
- We created journey maps to understand governance and decision making during all the stages of research in the two groups.
- We used a set of critical outcomes of research engagement as a framework to ground the work; however, it was difficult to entirely separate one outcome from the other.
- Our study design was appropriate for the exploratory nature of the study; however, we were unable to ensure that both groups were equally matched in terms of experience, skills and knowledge.

For peer review only - http://bmjopen.bmj.com/site/about/guidelines.xhtml

1. Introduction

There is a substantive body of work reporting the various ways in which patients are involved in the conduct and design of research,[1–3] and various frameworks and guidelines for supporting, evaluating and reporting patient engagement (PE) in research.[4,5] There are also studies showing the value of such engagement to the patient or researcher, such as a sense of purpose and being empowered; greater awareness of and appreciation for research; improved relationship with illness; feeling valued; and gaining new skills and knowledge.[6–8] There are fewer publications on how engagement impacts the research design, approach and outcomes.[6,9,10] This could be attributed to the lack of validated evaluation tools that are publicly available, informed by the literature and grounded in a theoretical or conceptual framework, inclusive of patient involvement in their development and reporting. [11–14] Some studies report hypothesized impacts instead of presenting evidence of impact.[8,15] None to our knowledge capture the impact across the whole research spectrum and outcomes.

We used observation methodology to obtain detailed and contextualized insights of the impact of research engagement throughout a health research study. This qualitative methodology has not been used extensively to study research engagement, likely due to analytical and practical challenges associated with studying a phenomenon thoroughly and at length.[16] Observational methods involve the systematic, detailed observation of behavior and communication[17] and has been used by researchers when other methods such as interviews or surveys alone cannot fully capture the context and phenomenon under study.[18–20] Observation provides an in-depth understanding of people's actions, roles and behavior[21,22] and identifies barriers and opportunities to more equal participation, shared decision-making, and shared understanding.[23]

For peer review only - http://bmjopen.bmj.com/site/about/guidelines.xhtml

In this exploratory study, we observed the roles of patient research partners (PRP) and other stakeholders in the research, and the influence PRPs had on the project design, approach and outcomes.

Method:

We used direct observation of two groups, a "Patient Research Partner led Group" (PLG), led by a PRP, and an "Academic Researcher led Group" (RLG), led by an academic researcher to observe similarities and differences in research engagement across the two groups.

We recruited PRPs (n=2), clinicians (n=2), and researchers (n=2) across Canada for each group. We identified participants through national network platforms (e.g. Strategy for Patient-Oriented Research, Inflammation, Microbiome, and Alimentation: Gastro-Intestinal and Neuropsychiatric Effects (SPOR IMAGINE) Network), [24] and study team contacts using maximum variation purposive sampling to recruit PRPs, and convenience sampling to recruit researchers and clinicians. PRPs and researchers were eligible to participate if they had basic knowledge and skills to conduct qualitative research acquired either through POR training, education or participating in health care research. Living with a chronic digestive condition such as IBD was also a requirement for PRPs. All recruited members completed a screening survey, which included select items from the Patient Centered Outcome Research Institute's Ways of Engaging- ENgagement ACtivity Tool (WE-ENACT)[25] and were then assigned to the PLG or the RLG, matching the two groups to the extent possible.

Both groups designed and conducted an exploratory qualitative preference project over a pre-determined seven-month period, addressing the same research question: "*What factors or attributes are important to patients with Inflammatory Bowel Disease (IBD) in considering*

For peer review only - http://bmjopen.bmj.com/site/about/guidelines.xhtml

treatment tapering of biologics?" This question served as context for studying the impact of engagement since there is no standard regimen for managing adults with IBD and little evidence on patient preferences regarding treatment decisions when considering biologic tapering.[26,27] Moreover, the engagement of patients in the development and design of preferences studies is recommended as good research practice.[28,29] While the structure of the two groups was the same except for the differences in the leads, the governance and decision making about conducting the research was left to each group.

Due to the COVID-19 pandemic, and the location of group members, observation of engagement was virtual. We assigned one study staff (NS and KB), skilled in qualitative research, per group to observe unobtrusively, documenting all exchanges of online meetings and emails among group members. The staff received training in the four questions of observation (what to observe, how to observe, how to preserve what is observed, how to tell what was observed).[16] Observation was overt. Group members were informed in the consent and at the first group meeting that they were being observed and all comments would be anonymized in the analysis. After the first meeting where staff introduced themselves, they faded into the background so members could act naturally while discussing the project. We believe these strategies helped put them at ease and not alter their behavior consciously.[30]

The staff kept rigorous notes of meeting discussions and email chats (descriptive data), and documented their thoughts and questions during and after the discussions (reflective data) using a semi-structured guide.[31] (Supplementary Table 1) For example, the staff documented what changes PRPs proposed that were made or not made and why or how the groups appropriately integrated group member suggestions. The meetings were audio recorded to verify observation notes for their

For peer review only - http://bmjopen.bmj.com/site/about/guidelines.xhtml

quality and trustworthiness. Ethical practices were followed such as assigning a unique study number on the collected data.[30-34]

We used Dillon's Critical Outcomes of Research Engagement (CORE) and measures as a grounding framework to assess engagement in the two groups.[35] The eleven potential outcomes and related measures were suitable for our study, covering a broad spectrum of the research design, approach and short and long-term outcomes of engagement.

The data from both groups were analyzed thematically in four steps using NVivo-12 software[36]: organized the data; sorted the data by critical outcomes, research stages and critical activities; created a journey map.[37,38] for each group member by "member types" (PRPs, researchers and clinicians) to understand how each member type influenced and impacted the project. Our journey map outlined all the activities each group member as a column heading with each member ID as rows to understand what they did at each critical research task and how they influenced the task. Lastly, we compared the journey maps of the researchers, PRPs and clinicians in the PLG with those in the RLG to identify similarities and differences between the two groups for a broader understanding of the impact and the critical outcomes of research engagement. (Figure 1,2)

For peer review only - http://bmjopen.bmj.com/site/about/guidelines.xhtml

Figure 1. Observation Data Analysis Steps

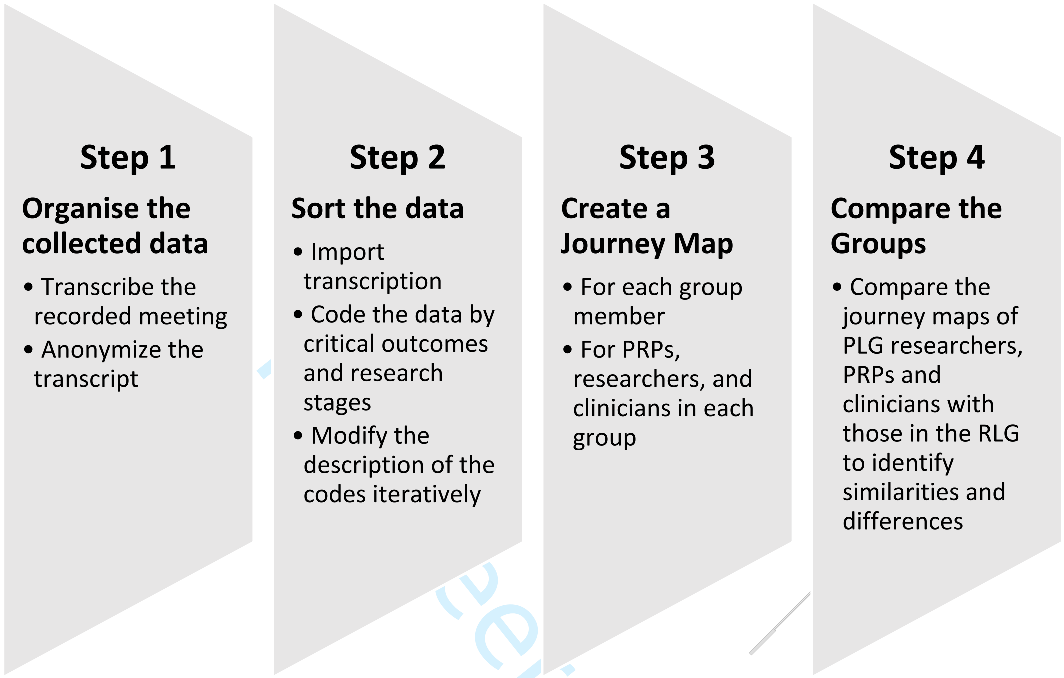

Two study staff (NS and KB) coded the data. A third coder (GM) discussed the coding separately with the other two coders, checked the quality of the coding, and ensured consistency between the two coders. NS and KB reflected on their personal values during the data collection and analysis process to identify any biases that may have affected the research, such as attachment bias to group members. We used this approach to facilitate good practice in coding and enhance the credibility of the analysis.[39,40]

We provided the two group leads training with a “baseline package” that was also available to all group members containing basic information about patient preference studies, qualitative research and high-level information about the project group work and deliverables. No other information was provided to either group.

For peer review only - http://bmjopen.bmj.com/site/about/guidelines.xhtml

Results

3.1. Study participants

Fourteen total participants were recruited for the two project groups from a pool of 29 eligible participants. The main reasons for non-participation were workload issues and health concerns. The majority were 35 years old and over (PLG n=5; RLG n=6); women (PLG n=5; RLG n=5); white (PLG n=4; RLG n=6)); had a graduate, PhD or a professional degree (PLG n=5; RLG n=7); and had been involved in patient-oriented research (POR) for over a year (PLG n=6; RLG n=4). Nine (PLG n=3; RLG n=6) felt prepared to contribute to this study and seven (PLG n=; RLG n=4) indicated they had previously worked with or knew at least one member in their group before this project.

PRPs in both groups were trained in conducting research projects using qualitative methods through the Patient and Community Engagement Research (PaCER) program,[41-43] or through other education opportunities. All the researchers had qualitative research expertise; some with no IBD-specific knowledge. The clinicians were associated with the SPOR IMAGINE Network.[24,44]

3.2. Similarities and differences between project groups

We present the observation results from the virtual and email discussions by the eleven Critical Outcomes of Research Engagement (CORE)[35] operationalized in our study (Table 1). No new outcome was identified during our analysis. Operationalizing these outcomes was challenging as they were established for direct inquiry with study team members, with overlapping measures among the eleven outcomes. Patient-centeredness was central to all the

For peer review only - http://bmjopen.bmj.com/site/about/guidelines.xhtml

outcomes of research engagement but we tried to keep the measures independent of each other during synthesis.

Table 1. Critical Outcomes of Research Engagement and study measures

Critical outcomes	Measures
1) Patient Centered	How were PRPs engaged in, and influenced, each stage of research and critical research tasks?
2) Meaningful	Are the research method and outcomes reflective of, and outcomes relevant to, the community and all group members?
3) Team Collaboration	What is the group members' comfort level during discussions? Do all group members trust and respect each other? Are all group members clear about their roles on the project? Are PRPs and researchers given an opportunity to gain skills and knowledge in ways that work for them?
4) Understandable	Are study materials patient-friendly, understandable and written in a common/plain language? Are all group members comfortable with the written materials? Evaluate the reading level of the research documents. Was the data presented in an accessible, understandable way to all members? Is the overarching goal, study purpose and research question understandable by everyone?
5) Rigorous	Did the group appropriately integrate PRP suggestions without compromising rigor?
6) Integrity and Adaptable	Did PRPs propose any changes to the study design, methods, materials etc. that were made/not made? If not made, explain why.
7) Legitimate	To what degree was the sample or study population diverse and representative/ unbiased?
8) Feasible	Are research goals and methods realistic and feasible?
9) Ethical and transparent	Are all methods ethical, culturally safe, and patient-friendly? Is data/privacy protection more patient-centered and/or changed? Is honest transparent communication consistent throughout the project?
10) Timely	Is conduct of research and sharing information with all group members timely?
11) Sustainable	Is there a plan for sharing study findings? What role did PRPs play to disseminate study findings?

[1]Adapted from Dillon's Critical Outcomes of Research Engagement (CORE) and measures[35]

Representative quotes from both groups for each outcome are included to further illustrate the findings (Table 2).

For peer review only - http://bmjopen.bmj.com/site/about/guidelines.xhtml

1) Patient Centered

PRPS in both groups engaged in ways that shaped the project conduct and outcomes. They wore dual hats, of patients and researchers, and influenced the project wearing these dual hats. PRPs in both groups took on both advisory and operational roles. The PLG lead took on many operational roles and influenced more project-related decisions than the RLG lead.

The PLG lead delegated tasks to stakeholders in the group ensuring that the patient voice was embedded in all aspects of the research. Stakeholders volunteered to take roles in the RLG throughout the research process. PRPs and researchers in this group shared all project related tasks except during data analysis. The PRP in this group was not familiar with the analysis tools so the analysis defaulted to the researchers.

PRPs in both groups discussed the research question and project rationale and helped define 'tapering' for the purpose of their projects. The patient and clinician experience on both teams provided a deeper understanding of patient and clinician needs, which informed the design, approach, and conduct of the project. PRPs helped the group define who would most benefit from the research, inclusion-exclusion criteria, sample size, research focus and how to recruit participants**.**

The PRPs in both groups also engaged in the literature review process in different ways. The PLG lead helped identify MeSH terms for the medical librarian, reviewed the search output, and extracted the list of attributes from the literature. The other PRPs were blinded to the results. RLG PRPs reviewed some articles and extracted data. Most of the project materials were developed by the PLG lead, excluding the interview guides developed by the other two PLG

For peer review only - http://bmjopen.bmj.com/site/about/guidelines.xhtml

PRPs. The recruitment flyer, demographic questionnaire, and the clinician interview guide were developed by the two PRPs in the RLG; the remaining materials were developed by RLG researchers.

Recruitment was managed completely by the PLG lead and data collection by the two PLG PRPs. The clinician recruitment and data collection were managed by RLG PRPs, and the patient recruitment and data collection were managed by RLG researchers. The PLG lead and a PLG researcher conducted the analysis. A PRP in the RLG reviewed and agreed with the coding of one of the clinician transcripts. PRPs in both groups were involved in data interpretation. PRPs in both groups also participated in knowledge translation discussions and came up with potential ideas along with their group members to share project findings.

For peer review only

For peer review only - http://bmjopen.bmj.com/site/about/guidelines.xhtml

Figure 2. Comparative Journey Maps of PRPs in the PLG and RLG Illustrating Patient-Centeredness

Research Stage	Academic Researcher led Group	Patient Research Partner led Group
Getting to know your team	PRPs introduce themselves to the group.	**Members know each other & their position in the group.**
Deciding on how to work together	One PRP proposes having roles and tasks assigned ahead. The group discusses this strategy but does not formalize roles. Both PRPs **volunteer to tasks** during the different project stages.	PRPs propose additional strategies to communicate and agree with the final plan. Both accept the **roles assigned** to them by the lead.
Helping the study team understand what information is relevant to patients	PRPs share their **lived experiences especially with biologics.** One PRP has side effects and wants to stop taking biologics.	PRPs share their lived experiences. Discussed their experience, **not specifically with the treatment**.
Refining the study question	*PRPs question the definition of tapering and are not comfortable using the word when it was not an option for patients.* **They recommend finalizing the definition of tapering before moving on to next steps.** They look at ways tapering is defined in the literature, discuss, *and agree with the final question, direction and project title.*	*PRPs question what tapering means in the context of the study. They do not like using words such as tapering or withdrawal when discussing tapering.* Both PRPs suggest ways to refine the question and *agree with the final direction and project title.*
Designing the study	PRPs recommend data collection from **both clinicians and patients**, items to be included in the survey. *They recommend items to include in the screening questionnaire and identify questions to ask patients during the interviews.*	PRPs recommend including both UC and Crohn's **patients** in the sample, a ranking exercise after the interviews, an interprovincial lens, conducting interviews over focus groups and **blinding the literature review results from the members collecting data.** *They recommend items to include in the screening questionnaire and identify questions to ask patients during the interviews.*
Developing the study material	**One PRP develops the recruitment flyer, provides questions for both the patient and clinician interview guides, recommends language to be included in the consent, and provides content for the online surveys.** *One PRP develops the interview guides* for clinicians and provides feedback on the patient interview guide. **Both provide feedback on all the study materials.**	*PRPs develop guides* for the focus group and interviews.
Participating in the literature search	PRPs propose questions for the search. **PRPs review papers and extract data.** One PRP identifies papers useful to finalize the definition of tapering and inform the research design.	**PRPs are blinded to the results of the review.**
Training team members on how to recruit and work with patients	**No role.**	**PRPs conduct a mock session of the focus group.**
Finding patients to participate in the study	*PRPs propose platforms and strategies for recruitment. One* PRP was willing to use their connections to identify potential candidates and support recruitment of patients. **One PRP recruits clinician participants.**	*One PRP provides names of potential recruitment platforms.*
Data collection	*One PRP conducts interviews of all the clinicians.*	*PRPs conduct the focus group and interviews.* **They influence the group to drop the "ranking exercise" after the first focus group.**
Analysis and Reviewing results	*One PRP reviews the coded data of one clinician transcript and shares insights with the group.*	PRPs take on an advisory role during data analysis. *They review the analyzed data and agree that it resonates with what they heard during the data collection process.*

Key similarities between the groups are emphasized in orange, italicized text. Key differences are emphasized in blue, bold text.

For peer review only - http://bmjopen.bmj.com/site/about/guidelines.xhtml

2) Meaningful

PRPs in both groups shared their experience with IBD. However, RLG PRPs spent more time sharing and discussing their experience with biologics than the PLG PRPs. These discussions helped other group members better understand biologics and what aspects of withdrawal may be important to capture from their perspective, and how others with similar lived experiences may want to participate in the project. PRP and clinician insights, concerns and questions were helpful to each group in defining a meaningful research question.

The PRP experience in both groups also informed the project design, recruitment and data collection approaches and materials to ensure that the projects and their outputs were valuable to all group members. An RLG PRP shared the side effects she faced due to biologics and even though she was in her third year of remission, was not allowed to get off biologics. This conversation contributed to the group discussing the differences in interpretation of "tapering", the frequency, dosage, side effects and how that might influence the patient experience with biologics.

3) Collaboration

Members in each group worked collaboratively throughout their projects. The group leads made significant efforts to ensure all members had opportunities to contribute based on their individual strengths and interests. They listened to their group member suggestions, were flexible in their approach, and checked in with members to ensure everyone was happy with how the project was progressing.

Email discussions and decisions were more common in the RLG with 296 emails sent among group members, and revisited during meetings attended by most group members. The RLG met 14 times, one to three times per month, over the seven-month project period. The PLG

For peer review only - http://bmjopen.bmj.com/site/about/guidelines.xhtml

used emails predominately to share project updates and agendas. This group met weekly in the first two months of the project, for a total of 24 virtual meetings over the project period. Decisions were taken mainly during the meetings attended by most group members including one clinician. Irrespective of the approach, members in both groups shared ideas and opinions freely. Any differing views were resolved through respectful discussions.

Collaboration seemed easier in the PLG than in the RLG. No body cues indicated that participants were uneasy or unhappy with most decisions. Members got on the same page quickly and all were on-board with the final decisions. For example, there was a lot of conversation around the recruitment flyer in the PLG resulting in the unanimous decision to rephrase some language in the flyer. The meetings at the start of the project between the PLG lead and group members to discuss interests, availability, skills and desired roles to finalize the project plan; discussions on communication strategies; the lead taking on a time-intensive roles; frequent team meetings in the initial phases of the project; all seemed to contribute to the high degree of collaboration in this group.

There was a strong sense of teamwork at the start of the RLG's project but the lack of clarity around the project question caused frustration and disengagement, especially among the PRPs. The lead was not sufficiently knowledgeable about IBD to facilitate a decision. There were also conflicting opinions about the study design. Compromises were made by some RLG members on the final design. Unmet expectations and role ambiguity contributed to one PRP in this group withdrawing from the project. The lead had hoped that stakeholders would identify and take on opportunities where their input would be more helpful but this approach did not work. Commitment to the project and continued support of each other, especially from the remaining PRP, helped restore morale and sustained participation in the RLG.

For peer review only - http://bmjopen.bmj.com/site/about/guidelines.xhtml

Capacity building of PRPs also facilitated collaboration. For example, a PRP in the RLG was trained in interview facilitation by a researcher from that group. A PLG researcher described how framework analysis is carried out and supported the lead throughout this process. These two group members collaboratively sorted and refined the themes and identified attributes by alternating coding and then reviewing the results together.

4) Understandable

The PRP and clinician experience helped contextualize "tapering" and how to frame it in a research question. The PRPs, especially in the RLG, led the discussions during the early stages of the research because of their experiential knowledge base. Their concerns, informed by first-hand experience taking biologics, reinforced the need for a formal rapid search of the literature to better understand the project rationale and definition of "tapering". Following multiple discussions and the literature review, the RLG agreed that biologic tapering could refer to decreasing the dose, increasing the interval between infusions/injections, switching to another non-biologic medication, or stopping or discontinuing treatment. A patient-friendly project title was subsequently discussed and finalised in both groups.

The PRPs in both groups developed or reviewed the study materials using clear, engaging language that was agreeable to all members. They also developed or provided feedback on the data collection tools, ensuring information important to ask study participants was included in the guides. The results of the PLG literature review were presented in simple, understandable terms. The PLG lead influenced the presentation of final list of candidate attributes with the list presented in lay language. The RLG used more clinical/research language for some of their attributes.

5) Rigorous

For peer review only - http://bmjopen.bmj.com/site/about/guidelines.xhtml

Group members made collective decisions in all project stages, balancing scientific evidence with PRP insights. The PLG lead presented initial thoughts and other group members built on her ideas. Best-practices were considered and tweaked to ensure the patient perspective was reflected and predominant in the design. The PLG conducted an unstructured focus group prior to the formal interviews, alongside a literature review, to better grasp the patient perspective of biologics without being influenced by the literature. Many members felt this approach would add value without impacting the rigor of the project. A PLG researcher reiterated the importance of doing a rapid search of the tweaked approach to ensure the design was defensible. The PLG also discussed rigor during the literature review, recruitment, data collection and analysis.

The RLG followed a more evidence-based approach, conducting a formal rapid review of the literature and using the results to establish a clear context and foundation for the research question and project design. A PRP and a clinician provided a clear rationale for capturing data from both clinicians and patients, which was accepted by group members despite the patient-focused scope of the project.

Both groups used best practices when conducting, analyzing and reporting the results. The PLG collected data from one focus group and eight interviews and stopped data collection as no new themes were being identified. The RLG collected data from three clinician interviews and two patient interviews. The sample size of patient interviews was not realized as planned.

The analyzed data were shared with RLG members iteratively as it was being coded. The transcripts were double coded in both groups. A PLG researcher and the lead independently coded all the data. RLG researchers conducted most of the coding; however, the RLG PRP coded one clinician transcript. The researchers in both groups developed the initial priori framework to

guide the coding process. PRPs and clinicians in the two groups reviewed the list of themes to ensure nothing was missed or mis-represented and that the findings included the patient perspective.

6) Integrity and Adaptable

The PLG and RLG group members were flexible and continuously improved the project process if the changes were logical, verifiable, rigorous and ethical. Both groups embraced challenges and found new ways of meeting the project objective. Common challenges included the lack of clarity of the project purpose, unclear definition of "tapering", obtaining timely ethics approval, and identifying project participants. Additionally, the RLG dealt with the PRP withdrawal from the study. All members in this group responded well to the changing situation, spending additional time working through sticking points in the research question and project design.

The PRP insights in both groups strengthened the quality and trustworthiness of the data in real time. For example, a PLG PRP influenced the decision of dropping the "ranking exercise" after the interviews because they felt that this exercise would be meaningless given the small sample size and varied life experiences of patients.

The RLG PRPs influenced the decision to not incorporate clinician perspectives in the patient interviews as this approach did not capture all the nuances of the patient experience. Members in both groups were involved in data interpretation and in identifying the final candidate attributes, ensuring the research findings were appropriate and justifiable.

7) Legitimate

Diverse and experienced PRPs in both groups brought value into their projects' decision-making process and enhanced the understanding of biologic tapering from the patient

For peer review only - http://bmjopen.bmj.com/site/about/guidelines.xhtml

perspective. For example, leveraging personal experience, a PRP pointed out the importance of including both Crohn's disease and Ulcerative Colitis in the project.

The qualitative interview findings also confirmed the values of some PRPs about the importance of shared decision-making with their gastroenterologist and other health care providers on tapering biologics. PRPs in both groups ensured that the data gathered was relevant to them. The groups employed purposive sampling and screened participants to gain diverse and/or representative patient perspectives. The PRP and clinician experience helped decide the screening questions to capture balanced views. A varied group of qualitative project participants from the IBD community provided their perspectives, resulting in an increased understanding about patient preferences for biologic medications; the appeal and feasibility of tapering biologics; and the perceived benefits and risks of doing so. The PLG interviewed patients from many Canadian provinces, with mild, moderate or severe symptoms of either Crohn's disease or Ulcerative Colitis, with nearly half of them using multiple biologics. Even though the sample size was small, the RLG patient participants were of different genders and ages, but from the same province, with both using multiple biologics. The potential for participant bias was also considered in the decision-making process.

8) **Feasible**

Managing the workload on top of full-time jobs or coursework, and other responsibilities, especially within short timeframes, was challenging for many members in both groups. The leads took on many time-consuming tasks, making it easier for group members to participate in the research. Both groups discussed and debated the feasibility of various project designs and collaboratively came up with solutions to accomplish the project goal. Influenced by a PRP and clinician, the RLG decided to conduct clinician and patient interviews to address the complex

For peer review only - http://bmjopen.bmj.com/site/about/guidelines.xhtml

study question. The PLG also discussed various designs, but decided to interview patients to complete the project within the timeframe. Additional factors that worked well for the PLG were a clear plan for team communication, clarity about roles and responsibilities and regular team meetings with a full complement of project members. The PLG lead, and the RLG PRPs leveraged past connections within the IBD community to help with recruitment.

9) Ethical and transparent

Each group collaboratively solved ethical dilemmas such as privacy of participants during the zoom focus group sessions versus individual interviews; how to start an interview so that participants feel safe and secure to discuss their personal experiences; whether to put the honorarium amount on the recruitment flyer; how to recruit participants without putting them and the PRPs at risk, etc. PRPs as well as researchers in both groups were sensitive to ethics practices, particularly surrounding recruitment and data collection.

For example, the PLG did not recruit through gastroenterology clinics since gastroenterologists were not particularly interested in discussing biologic tapering with their patients. All information was transparent to group members in the recruitment materials shared with them. The whole process (the methodology, including the design, data collection, coding, analysis, and tools used in data analysis) was discussed and known to all members of both groups. PRPs in the RLG specifically shared the qualitative study results with their group participants to satisfy the goal of transparency. Engagement during analysis ensured that the patient perspective was transparent in the findings.

10) Timely

To ensure timeliness of the two projects, both leads took on many tasks. Virtual meetings and finding convenient times were a hurdle; the PLG ended up scheduling late-evening meetings.

For peer review only - http://bmjopen.bmj.com/site/about/guidelines.xhtml

Group commitment to project success, sharing of responsibilities and an interest in POR kept the two projects moving in a timely manner.

A feasible design enabled recruitment and data collection in the PLG. Prompt responses and constructive PLG meetings also contributed to timely decision making. Revisiting the project plan periodically was also helpful. The RLG spent substantial time during the early stages of the project building collaboration and coming to a consensus about the project question, which hindered the project timeline. The RLG lead recognized that completion of the project on time was important, but secondary to ensuring that all group members were happy with the research question and project design.

11) Sustainable

PE was visible throughout the research process and across various research tasks in both groups. All group members, including PRPs, had the necessary pre-requisites (training, exposure, and preparedness) for making decisions and engaging on the project until the end. No health episodes prevented sustained engagement. Immediately defining and distributing group member roles significantly contributed to the sustainability of the PLG project. The design, approach, materials, and results eventually met the needs and expectations of all members in both groups resulting in continued participation. Beyond sharing the results through publications and presentations, the two groups proposed future research topics such as developing and evaluating decision aids for shared decision making.

For peer review only - http://bmjopen.bmj.com/site/about/guidelines.xhtml

Table 2. Illustrative Quotes for Critical Outcomes of Research Engagement from observation of the two project groups

Critical Outcomes of Research Engagement in both groups	Illustrative quotes
**1) Patient Centered**<br>PRPs in the PLG and RLG were engaged in all the stages and critical tasks of their group's qualitative projects, on advisory and operational roles, and influenced the stages and tasks they were involved in.<br>The lived experience of the PRPs in the RLG informed more aspects of the project than the PLG PRPs. The PLG lead influenced more project-related decisions than the lead in the RLG.	"I just wanted to echo that wearing that kind of dual role in this myself, I think there are definitely a lot of overlaps that I can already see from a research perspective as well as a person experiencing it, so I think it'd be interesting to actually have the literature review and focus groups running simultaneously and some of us that are doing the focus group with the patients and then some of us that are doing the literature review at the end, we kind of merge the two together that way you're not biased by what we're finding in either source?" PRP influencing the design<br><br>"…it'd be interesting to hear about the different treatments they've experienced right? Did they first try steroids, or did they first try a special diet, or something else and how did they get to biologics and then things like what factors would influence your decision for example, like recovery time, hospitals stay, and what would make you feel more confident, and what information would you need from your doctor…" PRP influencing the content of the interview guide
**2) Meaningful research**<br>The research process and content were reflective of the shared experience, beliefs and values of the PRPs in both groups, and the two project outputs relevant to all study stakeholders in the groups. The patient engagement in both groups resulted in a meaningful research experience for all the stakeholders in the two groups, with the PLG stakeholders more satisfied with their experience. Members of both groups were satisfied with the research outputs.	"Do we want to try to get from as many provinces as possible, I think it would be good to have that lens. In my experience, I was diagnosed when I was in BC and I'm a resident of Ontario so it was a very complicated process because I was out of province my health insurance was actually like it was done so I couldn't get the coverage for any medications. I think there will be others who may have similar experiences, or maybe different experiences so it'll be interesting to see how that ranges province to province." PRP influencing the sampling criterion<br><br>"I think there is value add in having both Crohn and UC perspectives at the table when we're doing a focus group. Just from personal experience, my experience was 180 degrees different from what my sister experience so having that kind of dual lens might be helpful." PRP influencing the sampling criterion
**3) Team Collaboration**<br>Both groups were collaborative through the entirety of the qualitative project process. The group leads made significant efforts to ensure that all members had opportunities to contribute, based on their individual strengths and interests. The PLG met more frequently than the RLG, with decisions taken predominately during these meetings, while many decisions were taken by email and during the group meetings in the RLG. Both groups shared their views and insights. There was support for each other and appreciation/acknowledgement of work. Collaboration seemed easier in the PLG than in the RLG. There was a shared understanding of roles and unanimity in the PLG's final decisions, but not always in the RLG. PRPs in both groups had opportunities to gain new skills and learn from their engagement.	"I'm trying to figure out what people are interested in. What type of role anyone is interested in. I mean each person that's on the team will have a different appetite for how much they want to be updated, and how much do they want to be involved, more communication." PLG lead exploring stakeholder interest/roles for the project plan<br><br>"I just wanted to chime into and second what X (other PRP) pointed to from a patient perspective, I think this title works in terms of just capturing what we want in, and that is the people that are on biologics..." PRPs collaborating with the group on project title
**4) Understandable**<br>The PRP and clinician experience served to understand and contextualize "tapering" of biologics in both groups. PRPs developed or reviewed the study materials using language that was clear, engaging and agreeable to all the group members. The PLG presented their output in lay language while the RLG used more clinical/research language.	"I think if you use the word changing you're going to get all the people who are being forced to change to biosimilars right now. Like if we put that in our title, I think we're going to get the wrong people." PRP reviewing the project materials to ensure an understandable project title<br><br>"The clinician … used a lot of terms, especially… medical abbreviations … so when it comes to transcribing those terms, I will be willing to provide the input. I mostly knew what he was saying there, there were a couple that I'm not familiar with, but can bounce that off you (clinician in the group)." PRP reviewing transcripts to ensure understandable data
**5) Rigorous**<br>Throughout all stages of the two group projects, group members took collective decisions, balancing scientific evidence with group member insights. The RLG took a more evidence-based approach while designing the project. The two study groups integrated group member suggestions into the design, approach and conduct of the two qualitative projects without compromising project rigor.	"I think we are reaching the saturation point, plus this individual is similar in demographic that we already have. I think one or two things this individual might say, but is it going to change the whole direction of where our data lies, I doubt it!" Researcher confirming data saturation<br><br>"Tapering (definition) could be: Decrease of dose; Increase of interval between two infusions/injections; Discontinuation; Replacement by a 'lower' medication. I think it will be very important to clearly define these for participants – the attributes important to patients may vary depending on the type of tapering being considered." PRP influencing project design

For peer review only - http://bmjopen.bmj.com/site/about/guidelines.xhtml

6) **Integrity and Adaptable**<br>The PLG and RLG group members were flexible and continuously improved the project process if the changes were logical, verifiable, rigorous and ethical. Both groups embraced challenges and found new ways of meeting the project objective. PRPs in both groups were involved in interpreting the data and in identifying the final candidate attributes, ensuring that the research findings were appropriate and justifiable.	"I have not heard of biologic tapering happening, and when I've talked to my GI about moving off the biologic somehow, he's super uncomfortable because from what I understand, and maybe the research has changed, the risk of recurrence is really when people have gone off. So, I think it's really important to understand what is meant by tapering in this context and the research that's available to support tapering." PRP influencing the group to study tapering in more depth before designing the project<br><br>"I think we need to drop the ranking exercise (based on what was heard during the first focus group), the ranking would be heavily influenced based on the life experiences that the person had, so depending on who's doing the ranking, the ranking could be skewed and I think it would be difficult for it to be representative of a larger population..." PRP suggests dropping the ranking exercise after conducting the first focus group
7) **Legitimate**<br>Diverse and experienced PRPs in the two groups brought value into their project's decision-making process and enhanced the understanding of tapering of biologics from the patient perspective. There was diverse representation of project participants in the qualitative projects of both groups, though the sample size was small in the RLG. Both groups considered how bias might impact their recruitment.	"a lot of the responses (about who can help make the decision about tapering) came back that it would be great information to get from my gastroenterologist. So, it wasn't like … I'd like to go online and do a Google search and get this information right at my fingertips … they wanted someone to relay that information to them." PRPs informing the group about the needs of diverse project participants<br><br>"just reflecting on the interviews, the categories seem logical to me, I feel it is pretty accurate. I actually like how it comes out, burden of disease, treatment, financial costs, coverage, I like that decision making- they talked about whether they used their healthcare provider or family or who else they might, like other patients" PRP confirming final list of attributes
8) **Feasible**<br>Members in both groups took on roles that were feasible for them. Collaboratively, they planned a project design and approach that was feasible to complete within the timeframe, without compromising the quality of the project. Time constraints experienced by the RLG negatively affected recruitment and data collection.	"For the study itself, due to time constraints and reflecting on the research question, I think we should focus solely on patient perspectives. We will definitely have to kind of brainstorm and look at the research that's been done before, to see what the best kinds of ways, or how it might be best to … gather their perspectives." PRP discussing the project design<br><br>"I struggle more to find participants for focus groups than for interviews. I think, for the longer part of the projects relying on multiple focus groups, in the world that we're in right now, might be just difficult to accomplish." PRP influencing the study approach
9) **Ethical and transparent**<br>PRPs in both groups collaboratively helped solve ethical dilemmas, and continuously checked assumptions of other group members during recruitment and data collection to ensure data collection materials and tools were transparent. Risks and potential harms to the patient were considered.	"I think it would be great to have the clinicians conducting the interviews, my question is would the interviewees be made aware of that?" PRP discussing risks and potential harms<br><br>"Are we trying to encourage people to do things that actually go against … clinical care guidelines." Clinician questions ethics
10) **Timely**<br>The PLG was able to complete their project within the stipulated timeframe, while the RLG spent substantial time defining the question, which prevented the group from completing data collection as planned. Members in both groups took collaborative decisions and made relevant changes in a timely manner.	"… do we have the time to also capture (patient blogs), because we're going to be starting the focus groups, we need to analyze we've got to write this thing up and it's all going to be done by the end of September, there's a lot of work there ahead of us, so … I don't think it's wrong to not include personal blogs if everyone agrees…" Researcher discussing feasibility<br><br>"…the point of the project is for the group to design something that reflects their ideas and what is important to them, so I actually think it is more important to get the design right than to get it done (on time)." RLG lead encourages group to spend more time on research question and design
11) **Sustainable**<br>The research addressed most members' needs and expectations, resulting in continued participation on the project. One PRP dropped out of the RLG due in part to unmet expectations. The key outputs met all group member's requirements in the two groups. The PLG offered to present project findings at conferences and workshops and considered publishing their engagement experience. Both groups also proposed future research topics.	"I'm glad I had an opportunity to review some of the literature in detail. I particularly appreciated reading more about dose reduction, dose cycling, and personalized approaches to tapering – I had always considered tapering as 'discontinuing' altogether, so these expanded concepts related to tapering were really neat to consider..." PRP<br><br>"feels good knowing all the members, and how accommodating everyone is to help out with the project." PLG Lead

For peer review only - http://bmjopen.bmj.com/site/about/guidelines.xhtml

4. Discussion

Using observation, we were able to comprehensively measure the impact of engagement across the research spectrum, and obtain contextualized insights of engagement in the two groups. We were able to gain a better understanding of the key ingredients to successful engagement; the influence PRPs had on the project design, approach and outcomes not captured through surveys; and operationalized the Critical Outcomes of Research Engagement. For example, we observed how the working partnership ensured transparency or fairness in the projects or what changes PRPs proposed that were made/not made and why. We also identified ways the two groups appropriately integrated group member suggestions without compromising project rigor. This study enriches existing literature using the observation method to assess research engagement, teasing out the input and influence of PRPs. While previous research has used methods such as surveys, interviews and focus groups to study engagement, the current study demonstrates that observation can be an effective method, provided the expertise to conduct and record the observations and resources are available[45].

The PRP experience on the projects was not tokenistic[44] - they engaged in multiple ways across the research phases ranging from sharing their experience to co-producing research[46-49]. They helped operationalize the research question; developed the project design and approach; conducted or participated in the literature review; collected data; and analyzed data or provided input in the analysis and interpretation of the results. As such, the project question, design, process, conduct and outcomes were reflective of their lived experience, beliefs and values. The research had value to many members in the groups and the PRPs felt valued as group members. No power imbalances[50] were observed, with members sharing ideas with each other throughout the project. Small talk at the start and close of meetings, positive and encouraging feedback from

For peer review only - http://bmjopen.bmj.com/site/about/guidelines.xhtml

the researchers and clinicians also made PRPs feel appreciated. These qualities are essential to nurture interpersonal relationships between group members.[51] Both projects were patient-centered, collaborative, meaningful, rigorous, adaptable, ethical, legitimate, understandable, feasible, timely and sustainable.

Consistent with the emerging literature, our results demonstrate that engagement can be sustained across the research spectrum and not limited to preliminary activities[52] provided there is adequate preparation and resources (i.e., funding, time)[53]; motivation at both the patient and researcher level[54]; training and supports for researchers to effectively engage with patients[54]; adequate training and supports of PRPs; and willingness of PRPs to take on roles in the later stages of a project[55]. PRPs in both groups had a high-level skills and training in POR and/or qualitative research, and could function both as researchers and patients, which is unusual in health research. Researchers also wore dual researcher and patient or researcher and clinician hats. The PLG acknowledged their dual roles and identities through “reflexivity”.[56] Studies have shown simple acknowledgement is insufficient, but concrete reflexive practices can help build trust, ensure transparency, authenticity and more rigorous research.[57,58]

The group leads were also vital in promoting engagement.[5] Previous studies suggest that the leads could be the main stumbling blocks to engagement if they lack the knowledge, skills, and experience on how best to do it, and do not possess the leadership qualities for collaborative work.[59,60] Our group leads were organized, communicative, respectful and committed, and regularly checked in or provided updates to group members. They “led” the operations of the project and “facilitated” engagement.

We also observed that relationship-building with PRPs in research takes time[61] and includes: a flexible engagement plan with clarity about roles and expectations, clarity about the

For peer review only - http://bmjopen.bmj.com/site/about/guidelines.xhtml

purpose and format of the collaboration, agreed goals, agreed communication strategies and ways to monitor project progress[3,46,62-66]. Core values that the diverse members bring to a projects should also be discussed for successful engagement, such as mutual respect and trust, equal partnerships, appreciation, compromise and support for each other.[64,65,67,68,69]

Our study was exploratory and would be difficult to replicate since it is not possible to control the myriad characteristics of the group members and the context. However, the findings of the study offer important insights into the value of engaging with PRPs in the context of patient preference studies. Future research examining engagement requires appropriate resourcing, and careful design to adequately address associated methodological challenges of observing and reporting engagement.

Ethics and dissemination:

All relevant ethics approvals were obtained prior to data collection from the University of Calgary [REB20-1563] and the University of British Columbia [H20-03385].

Acknowledgements:

The authors thank the 14 group members who participated in this study. They also gratefully acknowledge the support from study team members Aida Fernandes, Executive Director IMAGINE Network, Tracy Wasylak, Chief Program Officer, Strategic Clinical Networks™ with Alberta Health Services, Dr. Gilaad Kaplan, Gastroenterologist and Professor in the Cumming School of Medicine at the University of Calgary, and Louise Morrin, Senior Provincial Director, Medicine Strategic Clinical Network at Alberta Health Services during the different study phases. The authors also express their appreciation for the funders of this study.

For peer review only - http://bmjopen.bmj.com/site/about/guidelines.xhtml

References

1. Sacristán JA, Aguarón A, Avendaño-Solá C, et al. Patient involvement in clinical research: why, when, and how. *Patient Prefer Adherence*. 2016;10:631-640. doi:10.2147/PPA.S104259

2. McCarron TL, Clement F, Rasiah J, et al. Patients as partners in health research: A scoping review. *Health Expect*. 2021;24(4):1378-1390. doi:10.1111/hex.13272

3. Manafo E, Petermann L, Mason-Lai P, Vandall-Walker V. Patient engagement in Canada: a scoping review of the 'how' and 'what' of patient engagement in health research. *Health Res Policy Sys*. 2018;16(1):5. doi:10.1186/s12961-018-0282-4

4. Vanderhout S, Nicholls S, Monfaredi Z, et al. Facilitating and supporting the engagement of patients, families and caregivers in research: the "Ottawa model" for patient engagement in research. *Research Involvement and Engagement*. 2022;8(1):25. doi:10.1186/s40900-022-00350-0

5. Bellows M, Burns KK, Jackson K, Surgeoner B, Gallivan J. Meaningful and effective patient engagement: What matters most to stakeholders. *Patient Experience Journal*. 2015;2(1):18-28. doi:10.35680/2372-0247.1069

6. Merker VL, Hyde JK, Herbst A, et al. Evaluating the Impacts of Patient Engagement on Health Services Research Teams: Lessons from the Veteran Consulting Network. *J Gen Intern Med*. 2022;37(1):33-41. doi:10.1007/s11606-021-06987-z

7. Ashcroft J, Wykes T, Taylor J, Crowther A, Szmukler G. Impact on the individual: what do patients and carers gain, lose and expect from being involved in research? *Journal of Mental Health*. 2016;25(1):28-35. doi:10.3109/09638237.2015.1101424

8. Bhati DK, Fitzgerald M, Kendall C, Dahrouge S. Patients' engagement in primary care research: a case study in a Canadian context. *Res Involv Engagem*. 2020;6(1):65. doi:10.1186/s40900-020-00238-x

9. Harrington RL, Hanna ML, Oehrlein EM, et al. Defining Patient Engagement in Research: Results of a Systematic Review and Analysis: Report of the ISPOR Patient-Centered Special Interest Group. *Value in Health*. 2020;23(6):677-688. doi:10.1016/j.jval.2020.01.019

10. Vat LE, Warren M, Goold S, et al. Giving patients a voice: a participatory evaluation of patient engagement in Newfoundland and Labrador Health Research. *Res Involv Engagem*. 2020;6(1):39. doi:10.1186/s40900-020-00206-5

11. Fruytier SE, Vat LE, Camp R, et al. Monitoring and Evaluation of Patient Engagement in Health Product Research and Development: Co-Creating a Framework for Community Advisory Boards. *J Patient Cent Res Rev*. 2022;9(1):46-57. doi:10.17294/2330-0698.1859

12. Greenhalgh T, Hinton L, Finlay T, et al. Frameworks for supporting patient and public involvement in research: Systematic review and co-design pilot. *Health Expectations*. 2019;22(4):785-801. doi:10.1111/hex.12888

For peer review only - http://bmjopen.bmj.com/site/about/guidelines.xhtml

13. Vat LE, Finlay T, Jan Schuitmaker-Warnaar T, et al. Evaluating the "return on patient engagement initiatives" in medicines research and development: A literature review. *Health Expectations*. 2020;23(1):5-18. doi:10.1111/hex.12951

14. Boivin A, L'Espérance A, Gauvin FP, et al. Patient and public engagement in research and health system decision making: A systematic review of evaluation tools. *Health Expect*. 2018;21(6):1075-1084. doi:10.1111/hex.12804

15. Ball S, Harshfield A, Carpenter A, Bertscher A, Marjanovic S. *Patient and Public Involvement in Research: Enabling Meaningful Contributions*. RAND Corporation; 2019. doi:10.7249/RR2678

16. Bardon T, Garreau L, Abdallah C, Journé B, Korica M. Rethinking Observation: Challenges and Practices. *M@n@gement*. 2020;23. doi:10.37725/mgmt.v23i3.5562

17. Mays N, Pope C. Qualitative research: Observational methods in health care settings. *BMJ*. 1995;311(6998):182-184.

18. Kawulich BB. Participant Observation as a Data Collection Method. *Forum Qualitative Sozialforschung / Forum: Qualitative Social Research*. 2005;6(2). doi:10.17169/fqs-6.2.466

19. Salmon J. Using observational methods in nursing research. *Nurs Stand*. 2015;29(45):36-41. doi:10.7748/ns.29.45.36.e8721

20. Kawulich B. Collecting data through observation. In: ; 2012:150-160.

21. Walshe C, Ewing G, Griffiths J. Using observation as a data collection method to help understand patient and professional roles and actions in palliative care settings. *Palliative medicine*. 2011;26. doi:10.1177/0269216311432897

22. DeWalt KM, DeWalt BR. *Participant Observation: A Guide for Fieldworkers*. AltaMira Press; 2002.

23. Stevenson F. Observing interactions as an approach to understanding patients' experiences. In: Ziebland S, Coulter A, Calabrese JD, Locock L, eds. *Understanding and Using Health Experiences: Improving Patient Care*. Oxford University Press; 2013:0. doi:10.1093/acprof:oso/9780199665372.003.0004

24. Moayyedi P, MacQueen G, Bernstein CN, et al. IMAGINE Network's Mind And Gut Interactions Cohort (MAGIC) Study: a protocol for a prospective observational multicentre cohort study in inflammatory bowel disease and irritable bowel syndrome. *BMJ Open*. 2020;10(10):e041733. doi:10.1136/bmjopen-2020-041733

25. PCORI Engagement Activity Inventory (NET-ENACT AND WE-ENACT). Accessed August 10, 2022. https://ceppp.ca/en/evaluation-toolkit/pcori-engagement-activity-inventory-net-enact-and-we-enact/

26. Bressler B, Marshall JK, Bernstein CN, et al. Clinical Practice Guidelines for the Medical Management of Nonhospitalized Ulcerative Colitis: The Toronto Consensus. *Gastroenterology*. 2015;148(5):1035-1058.e3. doi:10.1053/j.gastro.2015.03.001

For peer review only - http://bmjopen.bmj.com/site/about/guidelines.xhtml

27. Panaccione R, Steinhart AH, Bressler B, et al. Canadian Association of Gastroenterology Clinical Practice Guideline for the Management of Luminal Crohn's Disease. *Journal of the Canadian Association of Gastroenterology*. 2019;2(3):e1-e34. doi:10.1093/jcag/gwz019

28. van Overbeeke E, Vanbinst I, Jimenez-Moreno AC, Huys I. Patient Centricity in Patient Preference Studies: The Patient Perspective. *Frontiers in Medicine*. 2020;7. Accessed August 2, 2022. https://www.frontiersin.org/articles/10.3389/fmed.2020.00093

29. Bridges JFP, de Bekker-Grob EW, Hauber AB, Heidenreich S, Janssen E, Bast A, Hanmer J, Danyliv A, Low E, Bouvy JC, Marshall DA. A roadmap for increasing the usefulness and impact of patient-preference studies in decision making in health: a good practices report of an ISPOR task force. Value Health, Feb 2023;26(2):153–162. doi: 10.1016/j.jval.2022.12.004.

30. Watts JH. Ethical and practical challenges of participant observation in sensitive health research. *null*. 2011;14(4):301-312. doi:10.1080/13645579.2010.517658

31. Roller MR. Facilitating Reflexivity in Observational Research: The Observation Guide & Grid. Research Design Review. Published April 26, 2016. Accessed August 8, 2022. https://researchdesignreview.com/2016/04/26/facilitating-reflexivity-in-observational-research-the-observation-guide-grid/

32. Roberts LD. Ethical Issues in Conducting Qualitative Research in Online Communities. *Qualitative Research in Psychology*. 2015;12(3):314-325. doi:10.1080/14780887.2015.1008909

33. Lopez-Dicastillo O, Belintxon M. The Challenges of Participant Observations of Cultural Encounters within an Ethnographic Study. *Procedia - Social and Behavioral Sciences*. 2014;132:522-526. doi:10.1016/j.sbspro.2014.04.347

34. Hine C. *Virtual Ethnography*. 1st edition. Sage Publications; 2000.

35. Dillon EC, Tuzzio L, Madrid S, Olden H, Greenlee RT. Measuring the Impact of Patient-Engaged Research: How a Methods Workshop Identified Critical Outcomes of Research Engagement. *J Patient Cent Res Rev*. 2017;4(4):237-246. doi:10.17294/2330-0698.1458

36. Best Qualitative Data Analysis Software for Researchers | NVivo. Accessed April 25, 2022. https://www.qsrinternational.com/nvivo-qualitative-data-analysis-software/home

37. Ly S, Runacres F, Poon P. Journey mapping as a novel approach to healthcare: a qualitative mixed methods study in palliative care. *BMC Health Services Research*. 2021;21(1):915. doi:10.1186/s12913-021-06934-y

38. Westbrook JI, Coiera EW, Sophie Gosling A, Braithwaite J. Critical incidents and journey mapping as techniques to evaluate the impact of online evidence retrieval systems on health care delivery and patient outcomes. *International Journal of Medical Informatics*. 2007;76(2):234-245. doi:10.1016/j.ijmedinf.2006.03.006

39. Braun V, Clarke V. Reflecting on reflexive thematic analysis. *Qualitative Research in Sport, Exercise and Health*. 2019;11(4):589-597. doi:10.1080/2159676X.2019.1628806

40. Watt D. On Becoming a Qualitative Researcher: The Value of Reflexivity. *TQR*. Published online January 15, 2015. doi:10.46743/2160-3715/2007.1645

For peer review only - http://bmjopen.bmj.com/site/about/guidelines.xhtml

For peer review only

41. PaCER - Patient and Community Engagement Research. PaCER - Patient and Community Engagement Research. Accessed April 25, 2022. https://pacerinnovates.ca

42. Shklarov S, Marshall DA, Wasylak T, Marlett NJ. "Part of the Team": Mapping the outcomes of training patients for new roles in health research and planning. *Health Expect*. 2017;20(6):1428-1436. doi:10.1111/hex.12591

43. Marlett N, Shklarov S, Marshall D, Santana MJ, Wasylak T. Building new roles and relationships in research: a model of patient engagement research. *Qual Life Res*. 2015;24(5):1057-1067. doi:10.1007/s11136-014-0845-y

44. IMAGINE - Imagine SPOR | Chronic Disease Network. IMAGINE. Accessed April 25, 2022. https://imaginespor.com/imagine-spor-2/

45. Paterson BL, Bottorff JL, Hewat R. Blending Observational Methods: Possibilities, Strategies, and Challenges. *International Journal of Qualitative Methods*. 2003;2(1):29-38. doi:10.1177/160940690300200103

46. Hahn DL, Hoffmann AE, Felzien M, LeMaster JW, Xu J, Fagnan LJ. Tokenism in patient engagement. *Fam Pract*. 2017;34(3):290-295. doi:10.1093/fampra/cmw097

47. Boaz A, Hanney S, Borst R, O'Shea A, Kok M. How to engage stakeholders in research: design principles to support improvement. *Health Research Policy and Systems*. 2018;16(1):60. doi:10.1186/s12961-018-0337-6

48. Garces JPD, Lopez GJP, Wang Z, et al. Eliciting Patient Perspective in Patient-Centered Outcomes Research: A Meta Narrative Systematic Review. *2012*.:164.

49. Majid U, Gagliardi A. Clarifying the degrees, modes, and muddles of "meaningful" patient engagement in health services planning and designing. *Patient Education and Counseling*. 2019;102(9):1581-1589. doi:10.1016/j.pec.2019.04.006

50. Concannon TW, Meissner P, Grunbaum JA, et al. A new taxonomy for stakeholder engagement in patient-centered outcomes research. *J Gen Intern Med*. 2012;27(8):985-991. doi:10.1007/s11606-012-2037-1

51. Ocloo J, Garfield S, Franklin BD, Dawson S. Exploring the theory, barriers and enablers for patient and public involvement across health, social care and patient safety: a systematic review of reviews. *Health Research Policy and Systems*. 2021;19(1):8. doi:10.1186/s12961-020-00644-3

52. Zibrowski E, Carr T, McDonald S, et al. A rapid realist review of patient engagement in patient-oriented research and health care system impacts: part one. *Research Involvement and Engagement*. 2021;7(1):72. doi:10.1186/s40900-021-00299-6

53. Finney Rutten LJ, Morris MA, Schrader LM, et al. Approaching patient engagement in research: what do patients with cardiovascular disease think? *Patient Prefer Adherence*. 2015;9:1061-1064. doi:10.2147/PPA.S84980

54. Manafo E, Petermann L, Mason-Lai P, Vandall-Walker V. Patient engagement in Canada: a scoping review of the 'how' and 'what' of patient engagement in health research. *Health Res Policy Sys*. 2018;16(1):5. doi:10.1186/s12961-018-0282-4

For peer review only - http://bmjopen.bmj.com/site/about/guidelines.xhtml

55. Crockett LK, Shimmin C, Wittmeier KDM, Sibley KM. Engaging patients and the public in Health Research: experiences, perceptions and training needs among Manitoba health researchers. *Research Involvement and Engagement*. 2019;5(1):28. doi:10.1186/s40900-019-0162-2

56. Government of Canada CI of HR. Ethics Guidance for Developing Partnerships with Patients and Researchers - CIHR. Published April 17, 2020. Accessed September 25, 2022. https://cihr-irsc.gc.ca/e/51910.html

57. Berger R. Now I see it, now I don't: researcher's position and reflexivity in qualitative research. *Qualitative Research*. 2015;15(2):219-234. doi:10.1177/1468794112468475

58. Geddis-Regan AR, Exley C, Taylor GD. Navigating the Dual Role of Clinician-Researcher in Qualitative Dental Research. *JDR Clinical & Translational Research*. 2022;7(2):215-217. doi:10.1177/2380084421998613

59. Olmos-Vega FM, Stalmeijer RE, Varpio L, Kahlke R. A practical guide to reflexivity in qualitative research: AMEE Guide No. 149. *Med Teach*. Published online April 7, 2022:1-11. doi:10.1080/0142159X.2022.2057287

60. Wicks P, Richards T, Denegri S, Godlee F. Patients' roles and rights in research. *BMJ*. 2018;362:k3193. doi:10.1136/bmj.k3193

61. Den Hartog DN, Koopman PL, Van Muijen JJ. Charismatic Leadership; A State of the Art. *Journal of Leadership Studies*. 1995;2(4):35-49. doi:10.1177/107179199500200404

62. Boden C, Edmonds AM, Porter T, et al. Patient partners' perspectives of meaningful engagement in synthesis reviews: A patient-oriented rapid review. *Health Expect*. 2021;24(4):1056-1071. doi:10.1111/hex.13279

63. Boaz A, Borst R, Kok M, O'Shea A. How far does an emphasis on stakeholder engagement and co-production in research present a threat to academic identity and autonomy? A prospective study across five European countries. *Research Evaluation*. 2021;30(3):361-369. doi:10.1093/reseval/rvab013

64. Etchegary H, Pike A, Patey AM, et al. Operationalizing a patient engagement plan for health research: Sharing a codesigned planning template from a national clinical trial. *Health Expectations*. 2022;25(2):697-711. doi:10.1111/hex.13417

65. Sheridan S, Schrandt S, Forsythe L, Hilliard TS, Paez KA. The PCORI Engagement Rubric: Promising Practices for Partnering in Research. *The Annals of Family Medicine*. 2017;15(2):165-170. doi:10.1370/afm.2042

66. Hovén E, Eriksson L, Månsson D'Souza Å, et al. What makes it work? Exploring experiences of patient research partners and researchers involved in a long-term co-creative research collaboration. *Research Involvement and Engagement*. 2020;6(1):33. doi:10.1186/s40900-020-00207-4

67. McCarron TL, Clement F, Rasiah J, Moffat K, Wasylak T, Santana MJ. Co-designing strategies to support patient partners during a scoping review and reflections on the process: a commentary. *Research Involvement and Engagement*. 2021;7(1):25. doi:10.1186/s40900-021-00272-3

For peer review only - http://bmjopen.bmj.com/site/about/guidelines.xhtml

68. Haywood K, Lyddiatt A, Brace-McDonnell SJ, Staniszewska S, Salek S. Establishing the values for patient engagement (PE) in health-related quality of life (HRQoL) research: an international, multiple-stakeholder perspective. *Qual Life Res*. 2017;26(6):1393-1404. doi:10.1007/s11136-016-1465-5

69. Bird M, Ouellette C, Whitmore C, et al. Preparing for patient partnership: A scoping review of patient partner engagement and evaluation in research. *Health Expect*. 2020;23(3):523-539. doi:10.1111/hex.13040

Authors' contributions:

DAM conceptualized the study and led the design, conduct and analysis of this study and the drafting of and revising of the article. DCL and SB conceptualized the study and led the design, conduct and analysis of this study and helped revise the manuscript. NL and KLB participated in the design, coordination, data collection, conduct and analysis of the study and in drafting and revising the manuscript. PM contributed to the acquisition and interpretation of data and reviewed the manuscript critically. All other authors participated in the design, conduct and analysis of the study and reviewed the manuscript critically. All authors approved the final version to be published and agreed to be accountable for all aspects of the work.

Funding statement:

This work was supported by the SPOR IMAGINE (Strategy for Patient-Oriented Research, Inflammation, Microbiome, and Alimentation: Gastro-Intestinal and Neuropsychiatric Effects) Network. The Network is supported by a grant from the Canadian Institute of Health Research (Funding Reference Number: 1715-000-001) with funding matched by McMaster University, University of Calgary, University of Alberta, Queen's University, Dalhousie University, Montreal Heart Institute Research Centre, Takeda Pharmaceutical Company, Allergan Incorporated, Alberta Innovates, Research Manitoba, Crohn's and Colitis Canada.

For peer review only - http://bmjopen.bmj.com/site/about/guidelines.xhtml

Competing interests statement:

The authors declared no potential conflicts of interest with respect to the research, authorship, and/or publication of this article.

Availability of data and material:

The ethics approval for this study does not support the sharing of raw data.

Word Count: 4757

For peer review only

Figure 1. Observation Data Analysis Steps

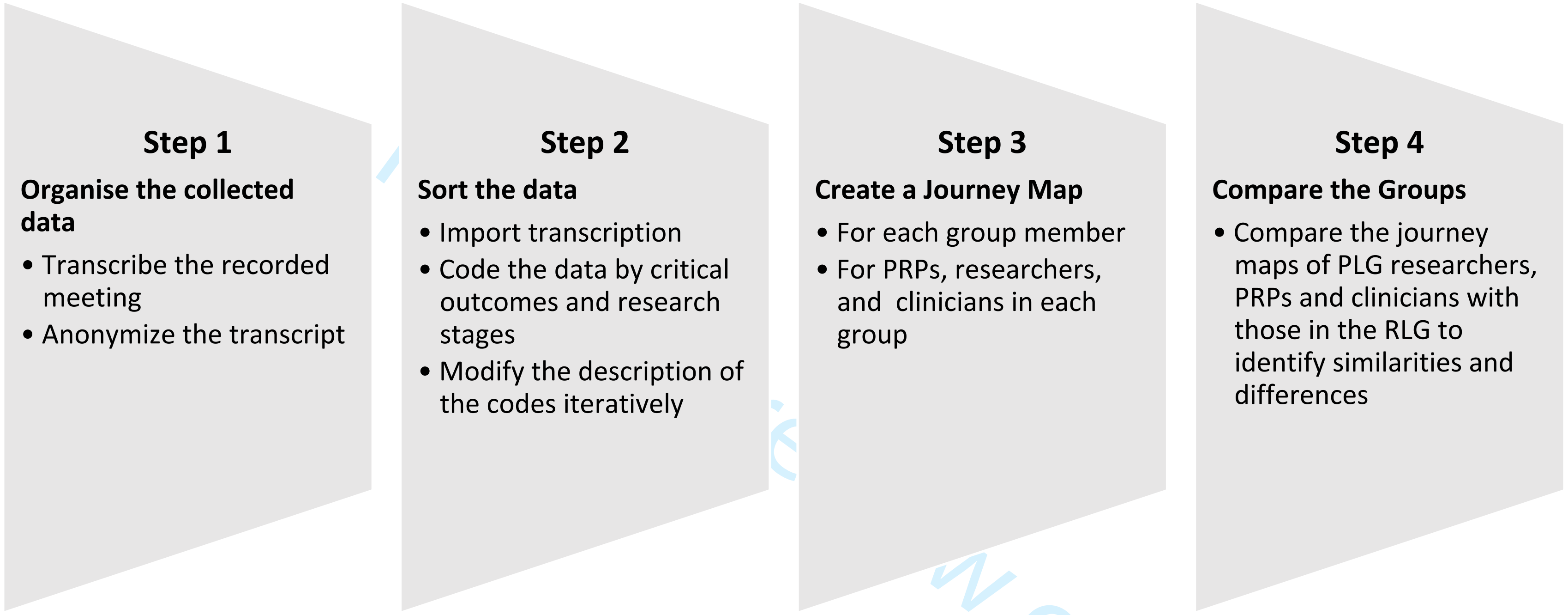

For peer review only - http://bmjopen.bmj.com/site/about/guidelines.xhtml

Figure 2. Comparative Journey Maps of PRPs in the PLG and RLG Illustrating Patient-Centeredness

**Research Stage**	**Academic Researcher led Group**	**Patient Research Partner led Group**
Getting to know your team	PRPs introduce themselves to the group.	**Members know each other & their position in the group.**
Deciding on how to work together	One PRP proposes having roles and tasks assigned ahead. The group discusses this strategy but does not formalize roles. Both PRPs **volunteer to tasks** during the different project stages.	PRPs propose additional strategies to communicate and agree with the final plan. Both accept the **roles assigned** to them by the lead.
Helping the study team understand what information is relevant to patients	PRPs share their **lived experiences especially with biologics.** One PRP has side effects and wants to stop taking biologics.	PRPs share their lived experiences. Discussed their experience, **not specifically with the treatment**.
Refining the study question	*PRPs question the definition of tapering and are not comfortable using the word when it was not an option for patients.* **They recommend finalizing the definition of tapering before moving on to next steps.** They look at ways tapering is defined in the literature, discuss, *and agree with the final question, direction and project title.*	*PRPs question what tapering means in the context of the study. They do not like using words such as tapering or withdrawal when discussing tapering.* Both PRPs suggest ways to refine the question and *agree with the final direction and project title.*
Designing the study	PRPs recommend data collection from **both clinicians and patients**, items to be included in the survey. *They recommend items to include in the screening questionnaire and identify questions to ask patients during the interviews.*	PRPs recommend including both UC and Crohn's **patients** in the sample, a ranking exercise after the interviews, an interprovincial lens, conducting interviews over focus groups and **blinding the literature review results from the members collecting data.** *They recommend items to include in the screening questionnaire and identify questions to ask patients during the interviews.*
Developing the study material	**One PRP develops the recruitment flyer, provides questions for both the patient and clinician interview guides, recommends language to be included in the consent, and provides content for the online surveys.** *One PRP develops the interview guides* for clinicians and provides feedback on the patient interview guide. **Both provide feedback on all the study materials.**	*PRPs develop guides* for the focus group and interviews.
Participating in the literature search	PRPs propose questions for the search. **PRPs review papers and extract data.** One PRP identifies papers useful to finalize the definition of tapering and inform the research design.	**PRPs are blinded to the results of the review.**
Training team members on how to recruit and work with patients	**No role.**	**PRPs conduct a mock session of the focus group.**
Finding patients to participate in the study	*PRPs propose platforms and strategies for recruitment. One* PRP was willing to use their connections to identify potential candidates and support recruitment of patients. **One PRP recruits clinician participants.**	*One PRP provides names of potential recruitment platforms.*
Data collection	*One PRP conducts interviews of all the clinicians.*	*PRPs conduct the focus group and interviews.* **They influence the group to drop the "ranking exercise" after the first focus group.**
Analysis and Reviewing results	*One PRP reviews the coded data of one clinician transcript and shares insights with the group.*	PRPs take on an advisory role during data analysis. *They review the analyzed data and agree that it resonates with what they heard during the data collection process.*

Key similarities between the groups are emphasized in orange, italicized text. Key differences are emphasized in blue, bold text.

For peer review only

Supplementary Table 1

Observation Guide Descriptive field notes for every project group activity

1) Meeting No:

2) Name of the Observer:

3) Group Observing:

4) Date of Observation:

5) Time of Observation: From………. To……………. Total meeting running time:

6) Meeting platform:

7) Number of group members:

8) Names of the group members: (Use ID numbers)

9) Time when each member joins and exists - (capturing how long each stays)

10) Group member roles in each activity during the different research stages and the interaction including the direction of communication, frequency of interaction of each group member, who took the decisions, etc.

- How does the meeting start? What's the mood in the room? Is it all business or do members chat with each other before or after meetings? Does the meeting start on time? Do all members seem to understand the purpose of the meeting, why they are there, and the agenda for the day? Are PRPs consulted when scheduling the activities?
- Does everyone speak or just some group members? Look for familiarity in the conversation for e.g. chatting about previous work together.
- Who is taking all the decisions during the discussion? At what stages/decisions are PRPs being consulted? Did PRPs propose any changes? Capture the changes proposed (changes in study design, methods, materials, etc.) and if not followed, the reasons why if

For peer review only

possible. Do the PRP have any influence on the final decision? Are group members supportive of other opinions in the group? Are there any instances of appreciation of work, giving credit openly during the discussions? Note down examples of members showing respect and supportive of the different viewpoints. Do PRPs initiate and take part in the discussions? How are the non-attendees informed of the decisions? If their input included in the final decision?

- Do PRPs lead any of the discussions? Did you observe silent moments in the conversation? Note: silences do not mean that the group member is uninterested or dis-engaged. It might mean reflective thinking. Jot down your thoughts, and rationale if you make this observation and how the lead/others involve the member and keep the conversation moving.
- Does anybody in the group use terms not understood by the rest of the group? Are there any written materials distributed prior to the meeting for review and questions? If yes, what were these materials? Document any discussions about the written materials for e.g. what does this medical word mean? What is the reading level of these documents?
- What training/support is provided to the PRPs and researchers in the group to help them contribute to the research? Are training requirements discussed? Is there any support request made to the main study team? Is the support provided and how?
- Do any conflicts emerge? What are they? How does the group respond and address the conflict? Note down any challenges that group members faced while engaging on this project.
- What does the morale of the group seem to be at the end of the meetings? Are "next steps" discussed? Are next steps understood? Does anybody on the group linger after the

For peer review only

meeting has closed to talk or do they leave immediately? What kinds of comments are stakeholders making as they leave?

Specific points to capture related to the launch phase (getting to know each other, working together and sharing experiences to help the group understand the supports PRP need)

- What strategy is employed by the group to get to know each? Does any member know another in their group prior to the first meeting? Does the study Lead introduce everyone or do the group members introduce themselves? Or Both?
- Does the group have a formal/informal plan to work together including a communication strategy, roles and responsibilities on the project?? Who developed this plan? Are PRPs involved in its development? If there is no plan, how did the group members operate?
- Did the PRPs in the group share their experience about living with IBD?

Specific points to capture related to the design phase (refining the research question, designing the study, conducting the literature review, developing the study material):

- Do all group members understand the research question, purpose of the study? What changes have been made to the research question? How did the group operationalize the research question? Are PRPs involved in influencing this decision? What is the result of any changes made?
- Who is involved in designing the research? What is each group members contributions to the study design? Are any changes proposed to the study design? Are all suggestions incorporated into the design? Are group members in agreement with the final decision/ validate the changes? Capture the changes made to design based on PRP input and the reason for changes not made. Are group members flexible in making changes keeping the

For peer review only

rigor of the study in mind? Is everyone on board with respect to the final design and approach?

- Is there any mention about the design being straightforward, and easy to accomplish within the time available? Is there any discussion and concern about the study goals and methods? For e.g., need for additional resources like time, expertise etc. to make the study happen. How are these addressed?
- Does the qualitative study design, data collection and analysis accommodate and show respect for participant diversity? Describe the study participants from the conversations.
- Does the group have discussions regarding data/privacy protection?
- What is the role of PRPs in reviewing the literature?
- Who developed the study material? What is the PRP input in their development? Document the materials developed/reviewed by PRP, changes made and reason for those not made. Are the materials written in lay audience language? Evaluate the reading level of the materials.
- Document discussions of personal benefit and benefit to the IBD larger community (for e.g. gained deeper understanding of biologics, gained skills/new knowledge through this engagement, contribute towards the advancement of POR)

Specific points to capture during the Implementation phase (recruitment, data collection, analysis)

- Who is involved in the recruitment of study participants? Who obtained consent/ screened the participants? What is the PRP input during this phase?
- Who is involved in data collection (patients, researchers or a combination of both)?

For peer review only

- Do the recruited study participants represent the living or lived experiences of actual patients, including groups that are typically under-represented in research?
- Who is involved in the analysis of the data? How are PRPs involved in the analysis?
- Is data collection transparent? Does the group use verifiable methods in performing the research and in reporting the results? Does the group pay attention to the ethical rules, regulations, guidelines while conducting the study? (for e.g., informed consent process, patient-friendly materials, data confidentiality and privacy etc.)
- How are the analysis and results shared with the group? Are the results shared with group members in a timely manner? Are the results validated by the PRP? Did they voice any concerns with the results and how were these addressed? Is the study completed within the stipulated time?

Specific points to capture during the Knowledge Translation (KT) Phase (explaining or applying results to real world setting, sharing study findings)

- Are there any discussions or plans about KT? What is the PRP's input during this phase? Are the PRPs in agreement with the plans?

Reflective field notes for every group engagement activity

Document the following elements intended to contextualize what you have observed based on your perspective.

- Note ideas, impressions, thoughts about what you observed. Include insights about why you believe specific phenomenon occurred.
- Include any unanswered questions or concerns that you think are important to record for future observations.

For peer review only

- Record anything that comes to mind that has not been captured elsewhere.

For peer review only

For peer review only

STROBE Statement—Checklist of items that should be included in reports of ***cohort studies***

	Item No	Recommendation	Page No
**Title and abstract**	1	(*a*) Indicate the study's design with a commonly used term in the title or the abstract<br>(*b*) Provide in the abstract an informative and balanced summary of what was done and what was found	1-2
**Introduction**			
Background/rationale	2	Explain the scientific background and rationale for the investigation being reported	3-4
Objectives	3	State specific objectives, including any prespecified hypotheses	3-4
**Methods**			
Study design	4	Present key elements of study design early in the paper	4-5
Setting	5	Describe the setting, locations, and relevant dates, including periods of recruitment, exposure, follow-up, and data collection	5-6
Participants	6	(*a*) Give the eligibility criteria, and the sources and methods of selection of participants. Describe methods of follow-up<br>(*b*) For matched studies, give matching criteria and number of exposed and unexposed	4
Variables	7	Clearly define all outcomes, exposures, predictors, potential confounders, and effect modifiers. Give diagnostic criteria, if applicable	6,9
Data sources/ measurement	8*	For each variable of interest, give sources of data and details of methods of assessment (measurement). Describe comparability of assessment methods if there is more than one group	6-9
Bias	9	Describe any efforts to address potential sources of bias	7
Study size	10	Explain how the study size was arrived at	4
Quantitative variables	11	Explain how quantitative variables were handled in the analyses. If applicable, describe which groupings were chosen and why	NA
Statistical methods	12	(*a*) Describe all statistical methods, including those used to control for confounding<br>(*b*) Describe any methods used to examine subgroups and interactions<br>(*c*) Explain how missing data were addressed<br>(*d*) If applicable, explain how loss to follow-up was addressed<br>(*e*) Describe any sensitivity analyses	NA
**Results**			
Participants	13*	(a) Report numbers of individuals at each stage of study—eg numbers potentially eligible, examined for eligibility, confirmed eligible, included in the study, completing follow-up, and analysed<br>(b) Give reasons for non-participation at each stage<br>(c) Consider use of a flow diagram	8
Descriptive data	14*	(a) Give characteristics of study participants (eg demographic, clinical, social) and information on exposures and potential confounders<br>(b) Indicate number of participants with missing data for each variable of interest<br>(c) Summarise follow-up time (eg, average and total amount)	8
Outcome data	15*	Report numbers of outcome events or summary measures over time	NA

For peer review only - http://bmjopen.bmj.com/site/about/guidelines.xhtml

Main results	16	(*a*) Give unadjusted estimates and, if applicable, confounder-adjusted estimates and their precision (eg, 95% confidence interval). Make clear which confounders were adjusted for and why they were included<br>(*b*) Report category boundaries when continuous variables were categorized<br>(*c*) If relevant, consider translating estimates of relative risk into absolute risk for a meaningful time period	8-27
Other analyses	17	Report other analyses done—eg analyses of subgroups and interactions, and sensitivity analyses	N/A
**Discussion**			
Key results	18	Summarise key results with reference to study objectives	27-28
Limitations	19	Discuss limitations of the study, taking into account sources of potential bias or imprecision. Discuss both direction and magnitude of any potential bias	2,30
Interpretation	20	Give a cautious overall interpretation of results considering objectives, limitations, multiplicity of analyses, results from similar studies, and other relevant evidence	28-29
Generalisability	21	Discuss the generalisability (external validity) of the study results	30
**Other information**			
Funding	22	Give the source of funding and the role of the funders for the present study and, if applicable, for the original study on which the present article is based	36

*Give information separately for exposed and unexposed groups.

Note: An Explanation and Elaboration article discusses each checklist item and gives methodological background and published examples of transparent reporting. The STROBE checklist is best used in conjunction with this article (freely available on the Web sites of PLoS Medicine at http://www.plosmedicine.org/, Annals of Internal Medicine at http://www.annals.org/, and Epidemiology at http://www.epidem.com/). Information on the STROBE Initiative is available at http://www.strobe-statement.org.

For peer review only - http://bmjopen.bmj.com/site/about/guidelines.xhtml

BMJ Open

Exploring the outcomes of research engagement using the observation method in an online setting

Journal:	*BMJ Open*
Manuscript ID	bmjopen-2023-073953.R1
Article Type:	Original research
Date Submitted by the Author:	02-Oct-2023
Complete List of Authors:	Marshall, Deborah; University of Calgary Department of Community Health Sciences; IMAGINE SPOR Chronic Disease Network<br>Suryaprakash, Nitya; The University of British Columbia School of Population and Public Health<br>Lavallee, Danielle; The University of British Columbia School of Population and Public Health; Michael Smith Health Research<br>Barker, Karis; University of Calgary Department of Community Health Sciences<br>Mackean, Gail; University of Calgary Department of Community Health Sciences<br>Zelinsky, Sandra; University of Calgary Department of Community Health Sciences; IMAGINE SPOR Chronic Disease Network<br>McCarron, Tamara; University of Calgary Department of Community Health Sciences<br>Santana, Maria-Jose; University of Calgary and Alberta Health Services Department of Paediatrics; Alberta SPOR SUPPORT Unit<br>Moayyedi, Paul; McMaster University Faculty of Health Sciences; IMAGINE SPOR Chronic Disease Network<br>Bryan, Stirling; The University of British Columbia School of Population and Public Health; British Columbia SPOR SUPPORT Unit
**Primary Subject Heading**:	Qualitative research
Secondary Subject Heading:	Health economics, Gastroenterology and hepatology, Patient-centred medicine
Keywords:	Patient Participation, QUALITATIVE RESEARCH, HEALTH ECONOMICS, GASTROENTEROLOGY, Adult gastroenterology < GASTROENTEROLOGY, Inflammatory bowel disease < GASTROENTEROLOGY

SCHOLARONE™
Manuscripts

I, the Submitting Author has the right to grant and does grant on behalf of all authors of the Work (as defined in the below author licence), an exclusive licence and/or a non-exclusive licence for contributions from authors who are: i) UK Crown employees; ii) where BMJ has agreed a CC-BY licence shall apply, and/or iii) in accordance with the terms applicable for US Federal Government officers or employees acting as part of their official duties; on a worldwide, perpetual, irrevocable, royalty-free basis to BMJ Publishing Group Ltd ("BMJ") its licensees and where the relevant Journal is co-owned by BMJ to the co-owners of the Journal, to publish the Work in this journal and any other BMJ products and to exploit all rights, as set out in our licence.

The Submitting Author accepts and understands that any supply made under these terms is made by BMJ to the Submitting Author unless you are acting as an employee on behalf of your employer or a postgraduate student of an affiliated institution which is paying any applicable article publishing charge ("APC") for Open Access articles. Where the Submitting Author wishes to make the Work available on an Open Access basis (and intends to pay the relevant APC), the terms of reuse of such Open Access shall be governed by a Creative Commons licence – details of these licences and which Creative Commons licence will apply to this Work are set out in our licence referred to above.

Other than as permitted in any relevant BMJ Author's Self Archiving Policies, I confirm this Work has not been accepted for publication elsewhere, is not being considered for publication elsewhere and does not duplicate material already published. I confirm all authors consent to publication of this Work and authorise the granting of this licence.

For peer review only

Exploring the outcomes of research engagement using the observation method in an online setting

Deborah A. Marshall,[1] Nitya Suryaprakash,[2] Danielle C. Lavallee,[3] Karis L. Barker,[1] Gail MacKean, [1] Sandra Zelinsky,[5] Tamara L. McCarron, [1] Maria J. Santana,[1,4] Paul Moayeddi,[6-7] and Stirling Bryan[2,3]

Affiliation:
1. Department of Community Health Sciences, University of Calgary, Calgary, Alberta, Canada
2. School of Population and Public Health, University of British Columbia, BC, Canada
3. Michael Smith Health Research, British Columbia, Vancouver, BC, Canada
4. Departments of Pediatrics, University of Calgary, Calgary, Alberta, Canada
5. Alberta, Strategy for Patient Oriented Research (SPOR) Support Unit, Alberta, Canada
6. IMAGINE SPOR Chronic Disease Network, Hamilton, Ontario, Canada.
7. Faculty of Health Sciences, McMaster University, Hamilton, Ontario, Canada.

Corresponding author(s):
Deborah A. Marshall, Cumming School of Medicine, University of Calgary, 3280 Hospital Drive NW, Health Research Innovation Centre (HRIC) Building, Room 3C58, Calgary, AB, T2N 4Z6, Canada. Email: damarsha@ucalgary.ca

ORCID IDs

Deborah A Marshall: https://orcid.org/0000-0002-8467-8008

Nitya Suryaprakash: https://orcid.org/0000-0001-8032-9129

Danielle C. Lavallee: https://orcid.org/0000-0002-5555-9675

Karis Barker: https://orcid.org/0000-0002-3530-566X

Gail MacKean: https://orcid.org/0000-0002-0209-4667

Sandra Zelinsky: https://orcid.org/0000-0001-5531-7660

Tamara L. McCarron: https://orcid.org/ 0000-0001-7242-1910

Maria J Santana: https://orcid.org/0000-0002-0202-5952

Stirling Bryan: https://orcid.org/0000-0001-7093-3058

For peer review only

Abstract

Objective: The objective of this study was to explore the outcomes of research engagement (PE) in the context of qualitative research.

Design: We observed engagement in two groups comprised of patients, clinicians and researchers tasked with conducting a qualitative preference exploration project in IBD. One group was led by a patient research partner (PLG) and the other by an academic researcher (RLG). A semi-structured guide and a set of critical outcomes of research engagement were used as a framework to ground our analysis.

Setting: The study was conducted online.

Participants: Patient research partners (n=5), researchers (n=5), and clinicians (n=4) participated in this study.

Main outcome measures: Transcripts of meetings, descriptive and reflective observation data of engagement during meetings and email correspondence between group members were analyzed to identify the outcomes of PE.

Results: Both projects were patient-centered, collaborative, meaningful, rigorous, adaptable, ethical, legitimate, understandable, feasible, timely and sustainable. Patient research partners (PRPs) in both groups wore dual hats as patients and researchers and influenced project decisions wearing both hats. They took on advisory and operational roles. Collaboration seemed easier in the PLG than in the RLG. The RLG PRPs spent more time than their counterparts in the PLG sharing their experience with biologics and helping their group identify a meaningful project question. A formal literature review informed the design, project materials and analysis in the

For peer review only - http://bmjopen.bmj.com/site/about/guidelines.xhtml

RLG while the formal review informed the project materials and analysis in the PLG. A PRP in the RLG and the PLG lead leveraged personal connections to facilitate recruitment. The outcomes of both projects were meaningful to all members of the group.

Conclusions: Our findings show that engagement of PRPs in research has a positive influence on the project design and delivery in the context of qualitative research in both the patient-led and researcher-led group.

Strengths and limitations of this study

- We used direct observation of research engagement, which provided a more robust understanding of PRP roles and influence on the research.
- Observation was in an online environment, and overt (group members were aware they were being directly observed in all project communications).
- We created journey maps to understand governance and decision making during all the stages of research in the two groups.
- We used a set of critical outcomes of research engagement as a framework to ground the work; however, it was difficult to entirely separate one outcome from the other.
- Our study design was appropriate for the exploratory nature of the study; however, we were unable to ensure that both groups were equally matched in terms of experience, skills and knowledge.

For peer review only - http://bmjopen.bmj.com/site/about/guidelines.xhtml

1. Introduction

There is a substantive body of work reporting the various ways in which patients are involved in the conduct and design of research,(1–3) and various frameworks and guidelines for supporting, evaluating and reporting patient engagement (PE) in research.(4,5) There are also studies showing the value of such engagement to the patient, such as a sense of purpose and being empowered; greater awareness of and appreciation for research; improved relationship with illness; feeling valued; and gaining new skills and knowledge.(6–8) There are fewer publications on the impact and outcomes of research engagement.(6,9,10) This could be attributed to the lack of validated evaluation tools that are publicly available, informed by the literature and grounded in a theoretical or conceptual framework, inclusive of patient involvement in their development and reporting.(11–14) Some studies report hypothesized impacts instead of presenting evidence of impact.(8,15) None to our knowledge capture the impact of PE across the whole research spectrum.

We used observation methodology to obtain detailed and contextualized insights of the impact of research engagement throughout a health research study. This qualitative methodology has not been used extensively to study research engagement, likely due to analytical and practical challenges associated with studying a phenomenon thoroughly and at length.(16) Observational methods involve the systematic, detailed observation of behavior and communication(17) and has been used by researchers when other methods such as interviews or surveys alone cannot fully capture the context and phenomenon under study.(18–20) Observation provides an in-depth understanding of people's actions, roles and behavior(21,22) and identifies barriers and opportunities to more equal participation, shared decision-making, and shared understanding.(23)

For peer review only - http://bmjopen.bmj.com/site/about/guidelines.xhtml

In this exploratory study, our objective was to explore the outcomes of research engagement in the context of qualitative research. We observed stakeholder - especially patient research partners (PRP) - engagement in two groups. Both groups designed and conducted an exploratory qualitative preference project over a pre-determined seven-month period, addressing the same research question: "*What factors or attributes are important to patients with Inflammatory Bowel Disease (IBD) in considering treatment tapering of biologics*?" We used this question as the context for studying the impact of engagement since there is no standard regimen for managing adults with IBD and little evidence on patient preferences regarding treatment decisions when considering biologic tapering.(24,25) Moreover, the engagement of patients in the development and design of preferences studies is recommended as good research practice.(26,27) We refer to the qualitative research conducted by the two groups as "projects" in this study.

2. Method

We used direct observation of two groups, a "Patient Research Partner led Group" (PLG), led by a PRP, and an "Academic Researcher led Group" (RLG), led by an academic researcher. Our rationale for studying two groups was to assess PE in two similar but distinctly different groups where PRPs would have sufficient opportunities to contribute and participate in the governance and decision making across the cycle of the group work. Our intention was not to judge the leads or the groups, but to look more broadly at how PRPs engage in and influence the group project work.

We recruited PRPs (n=2), clinicians (n=2), and researchers (n=2) across Canada for each group. We identified participants through national network platforms (e.g. Strategy for Patient-Oriented Research, Inflammation, Microbiome, and Alimentation: Gastro-Intestinal and

For peer review only - http://bmjopen.bmj.com/site/about/guidelines.xhtml

Neuropsychiatric Effects (SPOR IMAGINE) Network),(28) and study team contacts using maximum variation purposive sampling to recruit PRPs, and convenience sampling to recruit researchers and clinicians. PRPs and researchers were eligible to participate if they had basic knowledge and skills to conduct qualitative research acquired either through patient-oriented-research (POR) training, education or participating in health care research. Living with a chronic digestive condition such as IBD was also a requirement for PRPs. All recruited members completed a screening survey, which included select items from the Patient Centered Outcome Research Institute's Ways of Engaging- ENgagement ACtivity Tool (WE-ENACT)(29) and were then assigned to the PLG or the RLG, matching the two groups to the extent possible by their POR and qualitative research experience and training and demographics.

Due to the research taking place in 2021 during the COVID-19 pandemic and the location of group members, observation of engagement was virtual. We assigned one study staff (NS and KB) per group, skilled in qualitative research, to observe unobtrusively, documenting all exchanges of online meetings and emails among group members. The staff received training in the four questions of observation (what to observe, how to observe, how to preserve what is observed, how to tell what was observed).(16) The staff kept notes using a semi-structured guide(30) of the number of people involved in the discussions, the date of the discussion and the interactions and behaviors between group members (descriptive data). They also recorded their thoughts, biases, questions, initial interpretations of the discussions, potential themes, and direct quotes that seemed significant on a word document (reflective data) (Supplementary Table 1). For example, the staff documented what changes PRPs proposed that were made or not made and why or how the groups appropriately integrated group member suggestions. These notes were discussed during study team meetings to guide further data collection and generation.

For peer review only - http://bmjopen.bmj.com/site/about/guidelines.xhtml

All group meetings were audio recorded to verify observation notes and transcripts for their accuracy, quality and trustworthiness. The two staff listened to their group's recordings to ensure the transcripts were verbatim and their descriptive and reflective notes captured the non-verbal cues, the pre-defined themes and quotes accurately. A third staff performed oversight of this work at various points in the study and resolved discrepancies. Ethical practices were followed such as assigning a unique study number on all the transcripts of meetings, emails and descriptive and reflective notes.(31–34)

Observation was overt. Group members were informed in the consent and at the first group meeting that they were being observed and all data would be anonymized prior to the analysis. After the first meeting where staff introduced themselves, they faded into the background so members could act naturally while discussing the project. We believe these strategies helped put them at ease and not alter their behavior consciously.(31)

We used Dillon's Critical Outcomes of Research Engagement (CORE) and measures as a grounding framework to assess engagement in the two groups.(35) The eleven potential outcomes and related measures were suitable for our study, covering a broad spectrum of the research design, approach and short and long-term outcomes of engagement.

Table 1. Critical Outcomes of Research Engagement and study measures

Critical outcomes	Measures
1) Patient Centered	How were PRPs engaged in, and influenced, each stage of research and critical research tasks?
2) Meaningful	Are the research method and outcomes reflective of, and outcomes relevant to, the community and all group members?
3) Team Collaboration	What is the group members' comfort level during discussions? Do all group members trust and respect each other? Are all group members clear about their roles on the project? Are PRPs and researchers given an opportunity to gain skills and knowledge in ways that work for them?
4) Understandable	Are study materials patient-friendly, understandable and written in a common/plain language? Are all group members

For peer review only - http://bmjopen.bmj.com/site/about/guidelines.xhtml

	comfortable with the written materials? Evaluate the reading level of the research documents. Was the data presented in an accessible, understandable way to all members? Is the overarching goal, study purpose and research question understandable by everyone?
5) Rigorous	Did the group appropriately integrate PRP suggestions without compromising rigor?
6) Integrity and Adaptable	Did PRPs propose any changes to the study design, methods, materials etc. that were made/not made? If not made, explain why.
7) Legitimate	To what degree was the sample or study population diverse and representative/ unbiased?
8) Feasible	Are research goals and methods realistic and feasible?
9) Ethical and transparent	Are all methods ethical, culturally safe, and patient-friendly? Is data/privacy protection more patient-centered and/or changed? Is honest transparent communication consistent throughout the project?
10) Timely	Is conduct of research and sharing information with all group members timely?
11) Sustainable	Is there a plan for sharing study findings? What role did PRPs play to disseminate study findings?

[1]Adapted from Dillon's Critical Outcomes of Research Engagement (CORE) and measures.(35)

The data (transcripts of meetings, descriptive and reflective notes) from both groups were analyzed thematically in four steps using NVivo-12 software(36): 1) prepared and organized the data for analysis; 2) coded the data by critical outcomes, research stages and critical activities; 3) created a journey map(37,38) for each group member by "member types" (PRPs, researchers and clinicians) to understand how each member type influenced and impacted the project; and 4) compared the journey maps of all stakeholders especially the PRPs to identify the critical outcomes of PRP engagement in research. (Figure 1,2)

Data collection and analysis proceeded simultaneously using the CORE as *a priori* framework. Two study staff (NS and KB) coded their group data independently. A third staff (GM) coded some data from both groups at different stages of the project, merged their coding with NS or KB, discussed discrepancies and reached an agreement on the codes, sub codes and

For peer review only - http://bmjopen.bmj.com/site/about/guidelines.xhtml

their descriptions. Updated versions of the coding frame were shared between the two staff via the third staff and the data was recoded. After data collection was complete, the two staff created journey maps by stakeholder type for their respective groups. The staff reviewed the journey maps of both groups, and revisited the coding done to ensure that both agreed on the final journey maps. The journey maps of the patient-led group were compared to the research-led group maps to finalize the list of outcomes of research engagement. We held a virtual meeting with each group separately as a "member check-in exercise" to verify their results.

NS and KB reflected on their personal values during the data collection and analysis process to identify any biases that may have affected the research, such as attachment bias to group members. We used this approach to facilitate good practice in coding and enhance the credibility of the analysis.(39,40)

We provided the two group leads training about patient preference studies, qualitative research and about the project group work and deliverables. All this information was made available for use by other members of the two groups.

2.1. Patient and Public Involvement

Our study team included one PRP (SZ) living with Crohn's Disease who has extensive experience and training in conducting patient-oriented research on multi-disciplinary research teams. She is the Lead Patient Research Partner for the Alberta SPOR SUPPORT Unit and is a graduate of the Patient and Community Engagement Research (PaCER) program.(41–43) She was involved in the development of the research question and study design; finalizing the study approach and outcome measures; recruiting PLG and RLG group members; reviewing and providing feedback on the analyzed data; and reviewing this manuscript critically. We held an online meeting to discuss the results and outcomes of PE with members of both

For peer review only - http://bmjopen.bmj.com/site/about/guidelines.xhtml

groups. The group PRPs were also involved in all the stages and critical tasks of their respective qualitative projects.

3. Results

3.1. Study participants

Fourteen participants were recruited in total for the two project groups from a pool of 29 eligible participants. The main reasons for non-participation were workload issues and health concerns. The majority were 35 years old and over (PLG n=5; RLG n=6); women (PLG n=5; RLG n=5); white (PLG n=4; RLG n=6)); had a PhD or a professional degree (PLG n=3; RLG n=5); and had been involved in POR for over a year (PLG n=6; RLG n=4). Nine (PLG n=3; RLG n=6) felt prepared to contribute to this study and seven (PLG n=3; RLG n=4) indicated they had previously worked with or knew at least one member in their group before this project.

PRPs in both groups were trained in conducting research projects using qualitative methods through the PaCER program (41–43) or through other education opportunities. All the researchers had qualitative research expertise; some with no IBD-specific knowledge. The clinicians were affiliated with the SPOR IMAGINE Network.(28,44)

3.2. Critical outcomes of PRP engagement: similarities and differences of PRP engagement in the two groups

We present the observation results by the eleven Critical Outcomes of Research Engagement (CORE)(35) operationalized in our study (Table 1). No new outcome was identified during our analysis. Patient-centeredness was central to all the outcomes of research engagement but we tried to keep the measures independent of each other during synthesis. We gathered

For peer review only - http://bmjopen.bmj.com/site/about/guidelines.xhtml

information about all stakeholders but mainly focused on the contributions made by the PRPs in this paper. Representative quotes from both groups for each outcome are included to further illustrate the findings (Table 2).

1) Patient Centered

PRPs in both groups took on both advisory and operational roles. They influenced the project wearing dual hats of patients and researchers. The PLG lead took on many operational roles and influenced more project-related decisions than the RLG lead.

PRPs experience in the researcher-led group influenced the group to conduct a literature review first to finalize the research question. They reviewed articles along with researchers in their group, extracted data and helped their group identify papers useful to finalize the definition of tapering and study design. They also helped their team determine an optimal study design, recommended inclusion/exclusion criteria and data to collect such as duration of biologic use etc., provided a rationale for collecting data from clinicians and patients, identified questions to ask patients during the interviews, developed the clinician interview guides based on their experience, recruited and managed clinician recruitment and data collection and reviewed the coding of one of the clinician transcripts. Patient recruitment, data collection from patients and all the data analysis were managed by RLG researchers.

The PRPs in the patient-led group helped their group define 'tapering' for the purpose of their project. Unlike the PRPs in the RLG, they were not involved in the literature review process to avoid any bias in data collection. The patient lead conducted an informal search of the literature and proposed ideas for a project design and approach. The PRPs provided additional thoughts and structure to this design, some inclusion/exclusion criteria, variables to include in the screening questionnaire, and strategies to collect data. For example, one PRP

For peer review only - http://bmjopen.bmj.com/site/about/guidelines.xhtml

influenced the group to conduct a formal literature review simultaneous to the first focus group to have a draft list of attributes from the patient and clinician perspective that was used to inform the study materials and further data collection. The PRPs also developed the interview guides, conducted the interviews, and reviewed and confirmed the final themes.

PRPs in both groups were involved in data interpretation, in the knowledge translation discussions and came up with potential ideas along with their group members to share project findings.

2) Meaningful

PRPs in both groups were part of the decision-making processes during all the project stages, resulting in project deliverables that were relevant and meaningful to them and to the other stakeholders in the group. For example, the PRP experience in the RLG helped their group members better understand biologics and what aspects of withdrawal may be important to capture from their perspective. A PRP shared the side effects she faced due to biologics and even though she was in her third year of remission, was not allowed to get off biologics. This conversation contributed to the group discussing the differences in interpretation of "tapering", the frequency, dosage, side effects and how that might influence the patient experience with biologics. Even though not much was discussed specifically about treatment by the PRPs in the PLG, their experience provided insight into how others with similar lived experiences may want to participate in the study. A PRP shared her difficulty navigating insurance coverage for biologics between provinces, resulting in decisions about the inclusion/exclusion criteria of their project. The final list of attributes was discussed and finalized with the PRPs in both groups.

3) Collaboration

For peer review only - http://bmjopen.bmj.com/site/about/guidelines.xhtml

There was a strong sense of teamwork at the start of both group projects. However, collaboration seemed easier in the PLG than in the RLG which could be attributed to the clear roles that members had in the PLG; a clear plan for team communication; the lead taking on a number of time-intensive tasks; and frequent virtual meetings in the initial phases of the project with a full complement of project members.

Email discussions and decisions were more common in the RLG with the RLG meeting 14 times, over the seven-month project period. The PLG met weekly in the first two months of the project, for a total of 24 virtual meetings over the project period. Decisions were taken mainly during the meetings attended by most group members, including one clinician. Irrespective of the approach, members in both groups shared ideas and opinions freely and everyone's opinion was valued. There was small talk before and after meetings, and appreciative notes circulated and mentioned during meetings which made all stakeholders - especially the PRPs - feel appreciated.

Many collaborative decisions were taken by both groups during all project phases, impacting the process and results of the projects. For example, leveraging personal experience, a PRP in the PLG pointed out the importance of including both Crohn's disease and Ulcerative Colitis in the project. There were conflicting opinions about the study design in the RLG due to the lack of clarity around the project question. This caused frustration and disengagement especially among the PRPs. Many respectful dialogues were held to reach a consensus on the study design. The resultant design included data collection from both patients and clinicians. Another example of collaborative work was seen during the development of the study guides in both groups with input from PRPs and clinicians to ensure comprehensive data collection.

For peer review only - http://bmjopen.bmj.com/site/about/guidelines.xhtml

Capacity building of PRPs also facilitated collaboration and in turn impacted the results of the projects. For example, training of a PRPs in interview facilitation and conducting mock sessions enabled the PRPs to conduct some of the interviews in the RLG and all PLG interviews.

4) Understandable

The PRP and clinician experience helped contextualize "tapering" and how to frame it in a research question. A patient-friendly project title was subsequently discussed and finalised in both groups.

The PRPs in both groups developed or reviewed the study materials using clear, engaging language suitable for their project participants. They also developed or provided feedback on the data collection tools, ensuring information important to ask study participants was included in the guides and that the guides were easy to administer during data collection. For example, a PRP in the PLG simplified a question in the guide from "*What factors would make you feel confident that this is the right decision?" to "What would you like to know from your healthcare provider to make a decision*?" A PRP's query whether people can hypothetically think about going off biologics without actually wanting to go off biologics got the group rephrasing the introduction section of the guide.

The results of the PLG literature review were presented in simple, understandable terms. The PLG lead influenced the presentation of the final list of candidate attributes in lay language. The RLG used more clinical/research language for some of their attributes.

5) Rigorous

Group members made collective decisions in all project stages, balancing scientific evidence with PRP insights. The PLG lead presented initial thoughts and other group members built on her ideas. Wearing both a researcher and patient hat, a PRP proposed running focus

For peer review only - http://bmjopen.bmj.com/site/about/guidelines.xhtml

groups and a literature review simultaneously, blinding one to another to avoid biasing each other. Many members felt this approach would add value and rigor to the project. Subsequently, the patient-led group conducted an unstructured focus group prior to the formal interviews, alongside a literature review, to better grasp the patient perspective of biologics without being influenced by the literature.

The researcher-led group followed a more evidence-based approach, conducting a formal rapid review of the literature and using the results to establish a clear context and foundation for the research question and project design. A PRP and a clinician provided a clear rationale for capturing data from both clinicians and patients, which was accepted by group members despite the patient-focused scope of the project.

Both groups used best practices when conducting, analyzing and reporting the results. The PLG collected data from one focus group and eight interviews and stopped data collection as no new themes were being identified. The RLG collected data from three clinician interviews and two patient interviews. The sample size of patient interviews was not realized as planned. The analyzed data were shared with RLG members iteratively as it was being coded. The transcripts were double coded in both groups. PRPs and clinicians in the two groups validated the themes and ensured nothing was missed or mis-represented.

6) Integrity and Adaptable

The PLG and RLG group members were flexible and continuously improved the project process if the changes were logical, verifiable, rigorous and ethical. Both groups embraced challenges and found new ways of meeting the project objective. Common challenges included the lack of clarity of the project purpose, unclear definition of "tapering", obtaining timely ethics approval, and identifying project participants. Additionally, the RLG dealt with a PRP

For peer review only - http://bmjopen.bmj.com/site/about/guidelines.xhtml

withdrawal from the study due to a variety of reasons. All members in this group responded well to the changing situation, spending additional time working through sticking points in the research question and project design.

The PRP insights in both groups strengthened the quality and trustworthiness of the data in real time. For example, a PLG PRP influenced the decision of dropping the “ranking exercise” after the interviews because they felt that this exercise would be meaningless given the small sample size and varied life experiences of patients.

The RLG PRPs influenced the decision to not incorporate clinician perspectives in the patient interviews as this approach did not capture all the nuances of the patient experience. Members in both groups were involved in data interpretation and in identifying the final candidate attributes, ensuring the research findings were appropriate and justifiable.

7) Legitimate

Diverse and experienced PRPs in both groups brought value into their projects’ decision-making process and enhanced the understanding of the research question from the patient perspective. They also ensured that the approach and data gathered were relevant to them. The PRP and clinician experience in both groups helped decide the screening questions to capture diverse and/or representative patient perspectives. The PLG interviewed patients from many Canadian provinces, with mild, moderate or severe symptoms of either Crohn's disease or Ulcerative Colitis, with nearly half of them using multiple biologics. Even though the sample size was small, the RLG patient participants were of different genders and ages, but from the same province, with both using multiple biologics. Thus, a varied group of qualitative project participants from the IBD community provided their perspectives, resulting in an increased understanding about patient preferences for biologic medications; the appeal and feasibility of

For peer review only - http://bmjopen.bmj.com/site/about/guidelines.xhtml

tapering biologics; and the perceived benefits and risks of doing so. The qualitative interview findings also confirmed the values of some PRPs about the importance of shared decision-making with their gastroenterologist and other health care providers on tapering biologics

8) Feasible

Managing the workload on top of full-time jobs or coursework, and other responsibilities, especially within short timeframes, was challenging for many members in both groups. The leads took on many time-consuming tasks, making it easier for group members to participate in the research. Both groups discussed and debated the feasibility of various project designs and collaboratively came up with solutions to accomplish the project goal. Influenced by a PRP and clinician, the RLG decided to conduct clinician and patient interviews to address the complex study question. The PLG also discussed various designs, but decided to interview patients to complete the project within the timeframe. The PLG lead, and the RLG PRPs leveraged past connections within the IBD community to help with recruitment.

9) Ethical and transparent

Each group collaboratively solved ethical dilemmas such as privacy of participants during the zoom focus group sessions versus individual interviews; how to start an interview so that participants feel safe and secure to discuss their personal experiences; whether to put the honorarium amount on the recruitment flyer; how to recruit participants without putting them and the PRPs at risk, etc. PRPs as well as researchers in both groups were sensitive to ethics practices, particularly surrounding recruitment and data collection.

For example, the PLG did not recruit through gastroenterology clinics since gastroenterologists were not particularly interested in discussing biologic tapering with their patients. All information was transparent to group members in the recruitment materials shared

For peer review only - http://bmjopen.bmj.com/site/about/guidelines.xhtml

with them. The whole process (the methodology, including the design, data collection, coding, analysis, and tools used in data analysis) was discussed and known to all members of both groups. PRPs in the RLG specifically shared the qualitative study results with their group participants to satisfy the goal of transparency. Engagement during analysis ensured that the patient perspective was transparent in the findings.

10) Timely

To ensure timeliness of the two projects, both leads took on many tasks. Virtual meetings and finding convenient times were a hurdle; the PLG ended up scheduling late-evening meetings. Group commitment to project success, sharing of responsibilities and an interest in POR kept the two projects moving forward.

A feasible design enabled recruitment and data collection in the PLG. Prompt responses and constructive PLG meetings also contributed to timely decision making. Revisiting the project plan periodically was also helpful. The RLG spent substantial time during the early stages of the project building collaboration and coming to a consensus about the project question, which hindered the project timeline. The RLG lead recognized that completion of the project on time was important, but secondary to ensuring that all group members were happy with the research question and project design.

11) Sustainable

PE was visible throughout the research process and across various research tasks in both groups. All group members, including PRPs, had the necessary pre-requisites (training, exposure, and preparedness) for making decisions and engaging on the project until the end. No health episodes prevented sustained engagement. Immediately defining and distributing group member roles significantly contributed to the sustainability of the PLG project. The design, approach,

For peer review only - http://bmjopen.bmj.com/site/about/guidelines.xhtml

materials, and results eventually met the needs and expectations of all members in both groups, resulting in continued participation. Beyond sharing the results through publications and presentations, the two groups proposed future research topics such as developing and evaluating decision aids for shared decision making.

Table 2. Illustrative Quotes for Critical Outcomes of Research Engagement from observation of the two project groups

Critical Outcomes of Research Engagement in both groups	Illustrative quotes
**1) Patient Centered**<br>PRPs in the PLG and RLG were engaged in all the stages and critical tasks of their group's qualitative projects, on advisory and operational roles, and influenced the stages and tasks they were involved in.<br>The lived experience of the PRPs in the RLG informed more aspects of the project than the PLG PRPs. The PLG lead influenced more project-related decisions than the lead in the RLG.	"I just wanted to echo that wearing that kind of dual role in this myself, I think there are definitely a lot of overlaps that I can already see from a research perspective as well as a person experiencing it, so I think it'd be interesting to actually have the literature review and focus groups running simultaneously and some of us that are doing the focus group with the patients and then some of us that are doing the literature review at the end, we kind of merge the two together that way you're not biased by what we're finding in either source?" PRP influencing the design<br><br>"…it'd be interesting to hear about the different treatments they've experienced right? Did they first try steroids, or did they first try a special diet, or something else and how did they get to biologics and then things like what factors would influence your decision for example, like recovery time, hospitals stay, and what would make you feel more confident, and what information would you need from your doctor…" PRP influencing the content of the interview guide
**2) Meaningful research**<br>The research process and content were reflective of the shared experience, beliefs and values of the PRPs in both groups, and the two project outputs relevant to all study stakeholders in the groups. The patient engagement in both groups resulted in a meaningful research experience for all the stakeholders in the two groups, with the PLG stakeholders more satisfied with their experience. Members of both groups were satisfied with the research outputs.	"Do we want to try to get from as many provinces as possible, I think it would be good to have that lens. In my experience, I was diagnosed when I was in BC and I'm a resident of Ontario so it was a very complicated process because I was out of province my health insurance was actually like it was done so I couldn't get the coverage for any medications. I think there will be others who may have similar experiences, or maybe different experiences so it'll be interesting to see how that ranges province to province." PRP influencing the sampling criterion<br><br>"I think there is value add in having both Crohn and UC perspectives at the table when we're doing a focus group. Just from personal experience, my experience was 180 degrees different from what my sister experience so having that kind of dual lens might be helpful." PRP influencing the sampling criterion
**3) Team Collaboration**<br>Both groups were collaborative through the entirety of the qualitative project process. The group leads made significant efforts to ensure that all members had opportunities to contribute, based on their individual strengths and interests. The PLG met more frequently than the RLG, with decisions taken predominately during these meetings, while many decisions were taken by email and during the group meetings in the RLG. Both groups shared their views and insights. There was support for each other and appreciation/acknowledgement of work. Collaboration seemed easier in the PLG than in the RLG. There was a shared understanding of roles and unanimity in the PLG's final decisions, but not always in the RLG. PRPs in both groups had opportunities to gain new skills and learn from their engagement.	"I'm trying to figure out what people are interested in. What type of role anyone is interested in. I mean each person that's on the team will have a different appetite for how much they want to be updated, and how much do they want to be involved, more communication." PLG lead exploring stakeholder interest/roles for the project plan<br><br>"I just wanted to chime into and second what X (other PRP) pointed to from a patient perspective, I think this title works in terms of just capturing what we want in, and that is the people that are on biologics..." PRPs collaborating with the group on project title
**4) Understandable**<br>The PRP and clinician experience served to understand and contextualize "tapering" of biologics in both groups. PRPs developed or reviewed the study materials using language that was clear, engaging and agreeable to all the group members. The PLG presented their output in lay language while the RLG used more clinical/research language.	"I think if you use the word changing you're going to get all the people who are being forced to change to biosimilars right now. Like if we put that in our title, I think we're going to get the wrong people." PRP reviewing the project materials to ensure an understandable project title<br><br>"The clinician … used a lot of terms, especially… medical abbreviations … so when it comes to transcribing those terms, I will be willing to provide the input. I mostly knew what he was saying there, there were a couple that I'm not

For peer review only - http://bmjopen.bmj.com/site/about/guidelines.xhtml

	familiar with, but can bounce that off you (clinician in the group).” PRP reviewing transcripts to ensure understandable data
**5) Rigorous**<br>Throughout all stages of the two group projects, group members took collective decisions, balancing scientific evidence with group member insights. The RLG took a more evidence-based approach while designing the project. The two study groups integrated group member suggestions into the design, approach and conduct of the two qualitative projects without compromising project rigor.	“I think we are reaching the saturation point, plus this individual is similar in demographic that we already have. I think one or two things this individual might say, but is it going to change the whole direction of where our data lies, I doubt it!” Researcher confirming data saturation<br><br>“Tapering (definition) could be: Decrease of dose; Increase of interval between two infusions/injections; Discontinuation; Replacement by a 'lower' medication. I think it will be very important to clearly define these for participants – the attributes important to patients may vary depending on the type of tapering being considered.” PRP influencing project design
**6) Integrity and Adaptable**<br>The PLG and RLG group members were flexible and continuously improved the project process if the changes were logical, verifiable, rigorous and ethical. Both groups embraced challenges and found new ways of meeting the project objective. PRPs in both groups were involved in interpreting the data and in identifying the final candidate attributes, ensuring that the research findings were appropriate and justifiable.	“I have not heard of biologic tapering happening, and when I've talked to my GI about moving off the biologic somehow, he's super uncomfortable because from what I understand, and maybe the research has changed, the risk of recurrence is really when people have gone off. So, I think it's really important to understand what is meant by tapering in this context and the research that's available to support tapering.” PRP influencing the group to study tapering in more depth before designing the project<br><br>“I think we need to drop the ranking exercise (based on what was heard during the first focus group), the ranking would be heavily influenced based on the life experiences that the person had, so depending on who's doing the ranking, the ranking could be skewed and I think it would be difficult for it to be representative of a larger population...” PRP suggests dropping the ranking exercise after conducting the first focus group
**7) Legitimate**<br>Diverse and experienced PRPs in the two groups brought value into their project’s decision-making process and enhanced the understanding of tapering of biologics from the patient perspective. There was diverse representation of project participants in the qualitative projects of both groups, though the sample size was small in the RLG. Both groups considered how bias might impact their recruitment.	“a lot of the responses (about who can help make the decision about tapering) came back that it would be great information to get from my gastroenterologist. So, it wasn't like … I'd like to go online and do a Google search and get this information right at my fingertips … they wanted someone to relay that information to them.” PRPs informing the group about the needs of diverse project participants<br><br>“just reflecting on the interviews, the categories seem logical to me, I feel it is pretty accurate. I actually like how it comes out, burden of disease, treatment, financial costs, coverage, I like that decision making- they talked about whether they used their healthcare provider or family or who else they might, like other patients” PRP confirming final list of attributes
**8) Feasible**<br>Members in both groups took on roles that were feasible for them. Collaboratively, they planned a project design and approach that was feasible to complete within the timeframe, without compromising the quality of the project. Time constraints experienced by the RLG negatively affected recruitment and data collection.	“For the study itself, due to time constraints and reflecting on the research question, I think we should focus solely on patient perspectives. We will definitely have to kind of brainstorm and look at the research that's been done before, to see what the best kinds of ways, or how it might be best to … gather their perspectives.” PRP discussing the project design<br><br>“I struggle more to find participants for focus groups than for interviews. I think, for the longer part of the projects relying on multiple focus groups, in the world that we're in right now, might be just difficult to accomplish.” PRP influencing the study approach
**9) Ethical and transparent**<br>PRPs in both groups collaboratively helped solve ethical dilemmas, and continuously checked assumptions of other group members during recruitment and data collection to ensure data collection materials and tools were transparent. Risks and potential harms to the patient were considered.	“I think it would be great to have the clinicians conducting the interviews, my question is would the interviewees be made aware of that?” PRP discussing risks and potential harms<br><br>“Are we trying to encourage people to do things that actually go against … clinical care guidelines.” Clinician questions ethics
**10) Timely**<br>The PLG was able to complete their project within the stipulated timeframe, while the RLG spent substantial time defining the question, which prevented the group from completing data collection as planned. Members in both groups took collaborative decisions and made relevant changes in a timely manner.	“… do we have the time to also capture (patient blogs), because we're going to be starting the focus groups, we need to analyze we've got to write this thing up and it's all going to be done by the end of September, there's a lot of work there ahead of us, so … I don't think it's wrong to not include personal blogs if everyone agrees…” Researcher discussing feasibility<br><br>“…the point of the project is for the group to design something that reflects their ideas and what is important to them, so I actually think it is more important to get the design right than to get it done (on time).” RLG lead encourages group to spend more time on research question and design

For peer review only - http://bmjopen.bmj.com/site/about/guidelines.xhtml

**11) Sustainable**<br>The research addressed most members' needs and expectations, resulting in continued participation on the project. One PRP dropped out of the RLG due in part to unmet expectations. The key outputs met all group member's requirements in the two groups. The PLG offered to present project findings at conferences and workshops and considered publishing their engagement experience. Both groups also proposed future research topics.	"I'm glad I had an opportunity to review some of the literature in detail. I particularly appreciated reading more about dose reduction, dose cycling, and personalized approaches to tapering – I had always considered tapering as 'discontinuing' altogether, so these expanded concepts related to tapering were really neat to consider..." PRP<br><br>"feels good knowing all the members, and how accommodating everyone is to help out with the project." PLG Lead

Our results show that engagement of PRPs in research has a positive influence on the research design and delivery in the context of qualitative research in both the patient-led and researcher-led group. Using their lived experience, research knowledge and other life skills and experiences, PRPs in both groups helped operationalize the research question, the project design and approach; conducted or participated in the literature review; collected data; and analyzed data or provided input in the analysis and interpretation of the results. During the initial stages of the project, the PRPs in the RLG influenced their group to conduct a literature review first before finalizing the design. The PRPs in the PLG influenced their group to conduct the formal review simultaneously with the first focus group. They used the information to develop their study materials as well as during data analysis. The PRPs in the RLG influenced their group to collect information from both clinicians and patients. The PLG collected information from only patients. The final list of attributes was reviewed and finalized with the PRPs in both groups. As such, the research and the list of attributes were relevant and reflective of the lived experience beliefs and values of the PRPs in both groups. The stakeholders valued the experiences and knowledge that PRPs brought to the group. The resultant projects were patient-centered, collaborative, meaningful, rigorous, adaptable, ethical, legitimate, understandable, feasible, timely and sustainable.

4. Discussion

For peer review only - http://bmjopen.bmj.com/site/about/guidelines.xhtml

Using observation, we comprehensively measured the impact of engagement across the research spectrum and obtained contextualized insights of engagement in the two groups. We gained a better understanding of the key ingredients to successful engagement; the influence PRPs had on the research; and operationalized the Critical Outcomes of Research Engagement. For example, we observed how the working partnership ensured transparency or fairness in the projects or what changes PRPs proposed that were made/not made and why. We also identified ways the two groups appropriately integrated group member suggestions without compromising project rigor. This study enriches existing literature using the observation method to assess research engagement, teasing out the input and influence of PRPs. While previous research has used methods such as surveys, interviews and focus groups to study engagement, the current study demonstrates that observation can be an effective method, provided the expertise to conduct and record the observations and resources are available.(45)

The PRP experience on the projects was not tokenistic(46) - they engaged in multiple ways across the research phases ranging from sharing their experience to co-producing research.(47–50) No power imbalances(51,52) were observed. Members shared ideas with each other throughout the project. Informed decisions were made jointly through discussions. Small talk at the start and close of meetings, positive and encouraging feedback from the researchers and clinicians also made PRPs feel appreciated. These qualities are essential to nurture interpersonal relationships between group members.(52) Consistent with emerging literature, our results demonstrate that engagement can be sustained across the research spectrum and not limited to preliminary activities(53) provided there is adequate preparation and resources (i.e., funding, time)(54); motivation at both the patient and researcher level(3); training and supports for researchers to effectively engage with patients(3,54); adequate training and supports of

For peer review only - http://bmjopen.bmj.com/site/about/guidelines.xhtml

PRPs(54); and willingness of PRPs to take on roles in the later stages of a project.(55) PRPs in both groups had high-level skills and training in POR and/or qualitative research, and could function both as researchers and patients, which is unusual in health research. Some researchers also wore dual researcher and patient or researcher and clinician hats. The PLG acknowledged their dual roles and identities through "reflexivity".(56) Studies have shown simple acknowledgement is insufficient, but concrete reflexive practices can help build trust, ensure transparency, authenticity and more rigorous research.(57,58)

The group leads were also vital in promoting engagement.(5) Previous studies suggest that the leads could be the main stumbling blocks to engagement if they lack the knowledge, skills, and experience on how best to do it, and do not possess the leadership qualities for collaborative work.(59,60) Our group leads were organized, communicative, respectful and committed, and regularly checked in or provided updates to group members. They "led" the operations of the project and "facilitated" engagement.

We also observed that relationship-building with PRPs in research takes time(61) and includes: a flexible engagement plan with clarity about roles and expectations, clarity about the purpose and format of the collaboration, agreed goals, agreed communication strategies and ways to monitor project progress.(3,47,62–65) Core values that the diverse members bring to projects should also be discussed for successful engagement, such as mutual respect and trust, equal partnerships, appreciation, compromise and support for each other.(66–68)

Our study was exploratory and would be difficult to replicate since it is not possible to control the myriad characteristics of the group members and the context. Further, operationalizing these outcomes was challenging as they were established for direct inquiry with study team members, with overlapping measures among the eleven outcomes. However, the

For peer review only - http://bmjopen.bmj.com/site/about/guidelines.xhtml

findings of the study offer important insights into the value of engaging with PRPs in the context of patient preference studies. Future research using the observation methodology to examine outcomes of research engagement in other contexts and settings requires appropriate resourcing, and careful design to adequately address associated methodological challenges of observing and reporting engagement.

Acknowledgements:

The authors thank the 14 group members who participated in this study. They also gratefully acknowledge the support from study team members Aida Fernandes, Executive Director IMAGINE Network, Tracy Wasylak, Chief Program Officer, Strategic Clinical Networks™ with Alberta Health Services, Dr. Gilaad Kaplan, Gastroenterologist and Professor in the Cumming School of Medicine at the University of Calgary, and Louise Morrin, Senior Provincial Director, Medicine Strategic Clinical Network at Alberta Health Services during the different study phases. The authors also express their appreciation for the funders of this study.

Authors' contributions:

DAM, the guarantor, conceptualized the study and led the design, conduct and analysis of this study and the drafting of and revising of the article. DAM, DCL, and SB conceptualized the study and led the design, conduct and analysis of this study and helped revise the manuscript. NS and KLB participated in the design, coordination, data collection, conduct and analysis of the study and in drafting and revising the manuscript. PM contributed to the acquisition and interpretation of data and reviewed the manuscript critically. GM, SZ, TLM, and MJS participated in the design, conduct and analysis of the study and reviewed the manuscript

For peer review only - http://bmjopen.bmj.com/site/about/guidelines.xhtml

critically. All authors approved the final version to be published and agreed to be accountable for all aspects of the work.

Competing interests' statement:

Deborah A Marshall discloses consulting fees from the Office for Health Economics, Novartis, and Analytica during the conduct of this study. She also received support from Illumina for travel expenses to attend a meeting. Nitya Suryaprakash and Karis L Barker received reimbursement of expenses related to conference attendance from the SPOR IMAGINE Chronic Disease Network. All other authors declare no conflicts of interest relevant to the content of this article.

Funding statement:

This work was supported by the SPOR IMAGINE (Strategy for Patient-Oriented Research, Inflammation, Microbiome, and Alimentation: Gastro-Intestinal and Neuropsychiatric Effects) Network. The Network is supported by a grant from the Canadian Institute of Health Research (Funding Reference Number: 1715-000-001) with funding matched by McMaster University (Funding Reference Number: N/A), University of Calgary (Funding Reference Number: N/A), University of Alberta (Funding Reference Number: N/A), Queen's University (Funding Reference Number: N/A), Dalhousie University (Funding Reference Number: N/A), Montreal Heart Institute Research Centre (Funding Reference Number: N/A), Takeda Pharmaceutical Company (Funding Reference Number: N/A), Allergan Incorporated (Funding Reference Number: N/A), Alberta Innovates (Funding Reference Number: N/A), Research

For peer review only - http://bmjopen.bmj.com/site/about/guidelines.xhtml

Manitoba (Funding Reference Number: N/A), Crohn's and Colitis Canada (Funding Reference Number: N/A).

Availability of data and material:

The ethics approval for this study does not support the sharing of raw data.

Ethics and dissemination:

All relevant ethics approvals were obtained prior to data collection from the University of Calgary [REB20-1563] and the University of British Columbia [H20-03385].

References

1. Sacristán JA, Aguarón A, Avendaño-Solá C, et al. Patient involvement in clinical research: why, when, and how. *Patient Prefer Adherence*. 2016;10:631-640. doi:10.2147/PPA.S104259

2. McCarron TL, Clement F, Rasiah J, et al. Patients as partners in health research: A scoping review. *Health Expect*. 2021;24(4):1378-1390. doi:10.1111/hex.13272

3. Manafo E, Petermann L, Mason-Lai P, et al. Patient engagement in Canada: a scoping review of the 'how' and 'what' of patient engagement in health research. *Health Res Policy Sys*. 2018;16(1):5. doi:10.1186/s12961-018-0282-4

4. Vanderhout S, Nicholls S, Monfaredi Z, et al. Facilitating and supporting the engagement of patients, families and caregivers in research: the "Ottawa model" for patient engagement in research. *Research Involvement and Engagement*. 2022;8(1):25. doi:10.1186/s40900-022-00350-0

5. Bellows M, Burns KK, Jackson K, et al. Meaningful and effective patient engagement: What matters most to stakeholders. *Patient Experience Journal*. 2015;2(1):18-28. doi:10.35680/2372-0247.1069

6. Merker VL, Hyde JK, Herbst A, et al. Evaluating the Impacts of Patient Engagement on Health Services Research Teams: Lessons from the Veteran Consulting Network. *J Gen Intern Med*. 2022;37(1):33-41. doi:10.1007/s11606-021-06987-z

7. Ashcroft J, Wykes T, Taylor J, et al. Impact on the individual: what do patients and carers gain, lose and expect from being involved in research? *Journal of Mental Health*. 2016;25(1):28-35. doi:10.3109/09638237.2015.1101424

For peer review only - http://bmjopen.bmj.com/site/about/guidelines.xhtml

8. Bhati DK, Fitzgerald M, Kendall C, et al. Patients' engagement in primary care research: a case study in a Canadian context. *Res Involv Engagem*. 2020;6(1):65. doi:10.1186/s40900-020-00238-x

9. Harrington RL, Hanna ML, Oehrlein EM, et al. Defining Patient Engagement in Research: Results of a Systematic Review and Analysis: Report of the ISPOR Patient-Centered Special Interest Group. *Value in Health*. 2020;23(6):677-688. doi:10.1016/j.jval.2020.01.019

10. Vat LE, Warren M, Goold S, et al. Giving patients a voice: a participatory evaluation of patient engagement in Newfoundland and Labrador Health Research. *Res Involv Engagem*. 2020;6(1):39. doi:10.1186/s40900-020-00206-5

11. Fruytier SE, Vat LE, Camp R, et al. Monitoring and Evaluation of Patient Engagement in Health Product Research and Development: Co-Creating a Framework for Community Advisory Boards. *J Patient Cent Res Rev*. 2022;9(1):46-57. doi:10.17294/2330-0698.1859

12. Greenhalgh T, Hinton L, Finlay T, et al. Frameworks for supporting patient and public involvement in research: Systematic review and co-design pilot. *Health Expectations*. 2019;22(4):785-801. doi:10.1111/hex.12888

13. Vat LE, Finlay T, Jan Schuitmaker-Warnaar T, et al. Evaluating the "return on patient engagement initiatives" in medicines research and development: A literature review. *Health Expectations*. 2020;23(1):5-18. doi:10.1111/hex.12951

14. Boivin A, L'Espérance A, Gauvin FP, et al. Patient and public engagement in research and health system decision making: A systematic review of evaluation tools. *Health Expect*. 2018;21(6):1075-1084. doi:10.1111/hex.12804

15. Ball S, Harshfield A, Carpenter A, et al. *Patient and Public Involvement in Research: Enabling Meaningful Contributions*. RAND Corporation; 2019. doi:10.7249/RR2678

16. Bardon T, Garreau L, Abdallah C, et al. Rethinking Observation: Challenges and Practices. *M@n@gement*. 2020;23. doi:10.37725/mgmt.v23i3.5562

17. Mays N, Pope C. Qualitative research: Observational methods in health care settings. *BMJ*. 1995;311(6998):182-184.

18. Kawulich BB. Participant Observation as a Data Collection Method. *Forum Qualitative Sozialforschung / Forum: Qualitative Social Research*. 2005;6(2). doi:10.17169/fqs-6.2.466

19. Salmon J. Using observational methods in nursing research. *Nurs Stand*. 2015;29(45):36-41. doi:10.7748/ns.29.45.36.e8721

20. Kawulich B. Collecting data through observation. January 2012. In book: Doing Social Research: A global context (pp.150-160); McGraw-Hill Higher Education.

21. Walshe C, Ewing G, Griffiths J. Using observation as a data collection method to help understand patient and professional roles and actions in palliative care settings. *Palliative medicine*. 2011;26. doi:10.1177/0269216311432897

22. DeWalt KM, DeWalt BR. *Participant Observation: A Guide for Fieldworkers*. AltaMira Press; 2002.

For peer review only - http://bmjopen.bmj.com/site/about/guidelines.xhtml

23. Stevenson F. Observing interactions as an approach to understanding patients' experiences. In: Ziebland S, Coulter A, Calabrese JD, Locock L, eds. *Understanding and Using Health Experiences: Improving Patient Care*. Oxford University Press; 2013:0. doi:10.1093/acprof:oso/9780199665372.003.0004

24. Bressler B, Marshall JK, Bernstein CN, et al. Clinical Practice Guidelines for the Medical Management of Nonhospitalized Ulcerative Colitis: The Toronto Consensus. *Gastroenterology*. 2015;148(5):1035-1058.e3. doi:10.1053/j.gastro.2015.03.001

25. Panaccione R, Steinhart AH, Bressler B, et al. Canadian Association of Gastroenterology Clinical Practice Guideline for the Management of Luminal Crohn's Disease. *Journal of the Canadian Association of Gastroenterology*. 2019;2(3):e1-e34. doi:10.1093/jcag/gwz019

26. van Overbeeke E, Vanbinst I, Jimenez-Moreno AC, et al. Patient Centricity in Patient Preference Studies: The Patient Perspective. *Frontiers in Medicine*. 2020;7. Accessed August 2, 2022. https://www.frontiersin.org/articles/10.3389/fmed.2020.00093

27. Bridges JFP, de Bekker-Grob EW, Hauber B, et al. A Roadmap for Increasing the Usefulness and Impact of Patient-Preference Studies in Decision Making in Health: A Good Practices Report of an ISPOR Task Force. *Value Health*. 2023;26(2):153-162. doi:10.1016/j.jval.2022.12.004

28. Moayyedi P, MacQueen G, Bernstein CN, et al. IMAGINE Network's Mind And Gut Interactions Cohort (MAGIC) Study: a protocol for a prospective observational multicentre cohort study in inflammatory bowel disease and irritable bowel syndrome. *BMJ Open*. 2020;10(10):e041733. doi:10.1136/bmjopen-2020-041733

29. PCORI Engagement Activity Inventory (NET-ENACT AND WE-ENACT). Accessed August 10, 2022. https://ceppp.ca/en/evaluation-toolkit/pcori-engagement-activity-inventory-net-enact-and-we-enact/

30. Roller MR. Facilitating Reflexivity in Observational Research: The Observation Guide & Grid. Research Design Review. Published April 26, 2016. Accessed August 8, 2022. https://researchdesignreview.com/2016/04/26/facilitating-reflexivity-in-observational-research-the-observation-guide-grid/

31. Watts JH. Ethical and practical challenges of participant observation in sensitive health research. *null*. 2011;14(4):301-312. doi:10.1080/13645579.2010.517658

32. Roberts LD. Ethical Issues in Conducting Qualitative Research in Online Communities. *Qualitative Research in Psychology*. 2015;12(3):314-325. doi:10.1080/14780887.2015.1008909

33. Lopez-Dicastillo O, Belintxon M. The Challenges of Participant Observations of Cultural Encounters within an Ethnographic Study. *Procedia - Social and Behavioral Sciences*. 2014;132:522-526. doi:10.1016/j.sbspro.2014.04.347

34. Hine C. *Virtual Ethnography*. 1st edition. Sage Publications; 2000.

35. Dillon EC, Tuzzio L, Madrid S, et al. Measuring the Impact of Patient-Engaged Research: How a Methods Workshop Identified Critical Outcomes of Research Engagement. *J Patient Cent Res Rev*. 2017;4(4):237-246. doi:10.17294/2330-0698.1458

For peer review only - http://bmjopen.bmj.com/site/about/guidelines.xhtml

36. Best Qualitative Data Analysis Software for Researchers | NVivo. Accessed April 25, 2022. https://www.qsrinternational.com/nvivo-qualitative-data-analysis-software/home

37. Ly S, Runacres F, Poon P. Journey mapping as a novel approach to healthcare: a qualitative mixed methods study in palliative care. *BMC Health Services Research*. 2021;21(1):915. doi:10.1186/s12913-021-06934-y

38. Westbrook JI, Coiera EW, Sophie Gosling A, et al. Critical incidents and journey mapping as techniques to evaluate the impact of online evidence retrieval systems on health care delivery and patient outcomes. *International Journal of Medical Informatics*. 2007;76(2):234-245. doi:10.1016/j.ijmedinf.2006.03.006

39. Braun V, Clarke V. Reflecting on reflexive thematic analysis. *Qualitative Research in Sport, Exercise and Health*. 2019;11(4):589-597. doi:10.1080/2159676X.2019.1628806

40. Watt D. On Becoming a Qualitative Researcher: The Value of Reflexivity. *TQR*. Published online January 15, 2015. doi:10.46743/2160-3715/2007.1645

41. PaCER - Patient and Community Engagement Research. PaCER - Patient and Community Engagement Research. Accessed April 25, 2022. https://pacerinnovates.ca

42. Shklarov S, Marshall DA, Wasylak T, et al. "Part of the Team": Mapping the outcomes of training patients for new roles in health research and planning. *Health Expect*. 2017;20(6):1428-1436. doi:10.1111/hex.12591

43. Marlett N, Shklarov S, Marshall D, et al. Building new roles and relationships in research: a model of patient engagement research. *Qual Life Res*. 2015;24(5):1057-1067. doi:10.1007/s11136-014-0845-y

44. IMAGINE - Imagine SPOR | Chronic Disease Network. IMAGINE. Accessed April 25, 2022. https://imaginespor.com/imagine-spor-2/

45. Paterson BL, Bottorff JL, Hewat R. Blending Observational Methods: Possibilities, Strategies, and Challenges. *International Journal of Qualitative Methods*. 2003;2(1):29-38. doi:10.1177/160940690300200103

46. Hahn DL, Hoffmann AE, Felzien M, et al. Tokenism in patient engagement. *Fam Pract*. 2017;34(3):290-295. doi:10.1093/fampra/cmw097

47. Boaz A, Hanney S, Borst R, et al. How to engage stakeholders in research: design principles to support improvement. *Health Research Policy and Systems*. 2018;16(1):60. doi:10.1186/s12961-018-0337-6

48. Garces JPD, Lopez GJP, Wang Z, et al. Eliciting Patient Perspective in Patient-Centered Outcomes Research: A Meta Narrative Systematic Review. *2012.*:164.

49. Majid U, Gagliardi A. Clarifying the degrees, modes, and muddles of "meaningful" patient engagement in health services planning and designing. *Patient Education and Counseling*. 2019;102(9):1581-1589. doi:10.1016/j.pec.2019.04.006

For peer review only - http://bmjopen.bmj.com/site/about/guidelines.xhtml

50. Concannon TW, Meissner P, Grunbaum JA, et al. A new taxonomy for stakeholder engagement in patient-centered outcomes research. *J Gen Intern Med*. 2012;27(8):985-991. doi:10.1007/s11606-012-2037-1

51. Ocloo J, Garfield S, Franklin BD, et al. Exploring the theory, barriers and enablers for patient and public involvement across health, social care and patient safety: a systematic review of reviews. *Health Research Policy and Systems*. 2021;19(1):8. doi:10.1186/s12961-020-00644-3

52. Zibrowski E, Carr T, McDonald S, et al. A rapid realist review of patient engagement in patient-oriented research and health care system impacts: part one. *Research Involvement and Engagement*. 2021;7(1):72. doi:10.1186/s40900-021-00299-6

53. Finney Rutten LJ, Morris MA, Schrader LM, et al. Approaching patient engagement in research: what do patients with cardiovascular disease think? *Patient Prefer Adherence*. 2015;9:1061-1064. doi:10.2147/PPA.S84980

54. Crockett LK, Shimmin C, Wittmeier KDM, et al. Engaging patients and the public in Health Research: experiences, perceptions and training needs among Manitoba health researchers. *Research Involvement and Engagement*. 2019;5(1):28. doi:10.1186/s40900-019-0162-2

55. Government of Canada CI of HR. Ethics Guidance for Developing Partnerships with Patients and Researchers - CIHR. Published April 17, 2020. Accessed September 25, 2022. https://cihr-irsc.gc.ca/e/51910.html

56. Berger R. Now I see it, now I don't: researcher's position and reflexivity in qualitative research. *Qualitative Research*. 2015;15(2):219-234. doi:10.1177/1468794112468475

57. Geddis-Regan AR, Exley C, Taylor GD. Navigating the Dual Role of Clinician-Researcher in Qualitative Dental Research. *JDR Clinical & Translational Research*. 2022;7(2):215-217. doi:10.1177/2380084421998613

58. Olmos-Vega FM, Stalmeijer RE, Varpio L, et al. A practical guide to reflexivity in qualitative research: AMEE Guide No. 149. *Med Teach*. Published online April 7, 2022:1-11. doi:10.1080/0142159X.2022.2057287

59. Wicks P, Richards T, Denegri S, et al. Patients' roles and rights in research. *BMJ*. 2018;362:k3193. doi:10.1136/bmj.k3193

60. Den Hartog DN, Koopman PL, Van Muijen JJ. Charismatic Leadership; A State of the Art. *Journal of Leadership Studies*. 1995;2(4):35-49. doi:10.1177/107179199500200404

61. Boden C, Edmonds AM, Porter T, et al. Patient partners' perspectives of meaningful engagement in synthesis reviews: A patient-oriented rapid review. *Health Expect*. 2021;24(4):1056-1071. doi:10.1111/hex.13279

62. Boaz A, Borst R, Kok M, et al. How far does an emphasis on stakeholder engagement and co-production in research present a threat to academic identity and autonomy? A prospective study across five European countries. *Research Evaluation*. 2021;30(3):361-369. doi:10.1093/reseval/rvab013

For peer review only - http://bmjopen.bmj.com/site/about/guidelines.xhtml

63. Etchegary H, Pike A, Patey AM, et al. Operationalizing a patient engagement plan for health research: Sharing a codesigned planning template from a national clinical trial. *Health Expectations*. 2022;25(2):697-711. doi:10.1111/hex.13417

64. Sheridan S, Schrandt S, Forsythe L, et al. The PCORI Engagement Rubric: Promising Practices for Partnering in Research. *The Annals of Family Medicine*. 2017;15(2):165-170. doi:10.1370/afm.2042

65. Hovén E, Eriksson L, Månsson D'Souza Å, et al. What makes it work? Exploring experiences of patient research partners and researchers involved in a long-term co-creative research collaboration. *Research Involvement and Engagement*. 2020;6(1):33. doi:10.1186/s40900-020-00207-4

66. Haywood K, Lyddiatt A, Brace-McDonnell SJ, et al. Establishing the values for patient engagement (PE) in health-related quality of life (HRQoL) research: an international, multiple-stakeholder perspective. *Qual Life Res*. 2017;26(6):1393-1404. doi:10.1007/s11136-016-1465-5

67. Bird M, Ouellette C, Whitmore C, et al. Preparing for patient partnership: A scoping review of patient partner engagement and evaluation in research. *Health Expect*. 2020;23(3):523-539. doi:10.1111/hex.13040

68. McCarron TL, Clement F, Rasiah J, et al. Co-designing strategies to support patient partners during a scoping review and reflections on the process: a commentary. *Research Involvement and Engagement*. 2021;7(1):25. doi:10.1186/s40900-021-00272-3

Figure Legend:

Figure 1. Observation Data Analysis Steps.
Figure 2. Comparative Journey Maps of PRPs in the PLG and RLG Illustrating Patient-

Centeredness

Word Count: 4969

For peer review only

Figure 1. Observation Data Analysis Steps

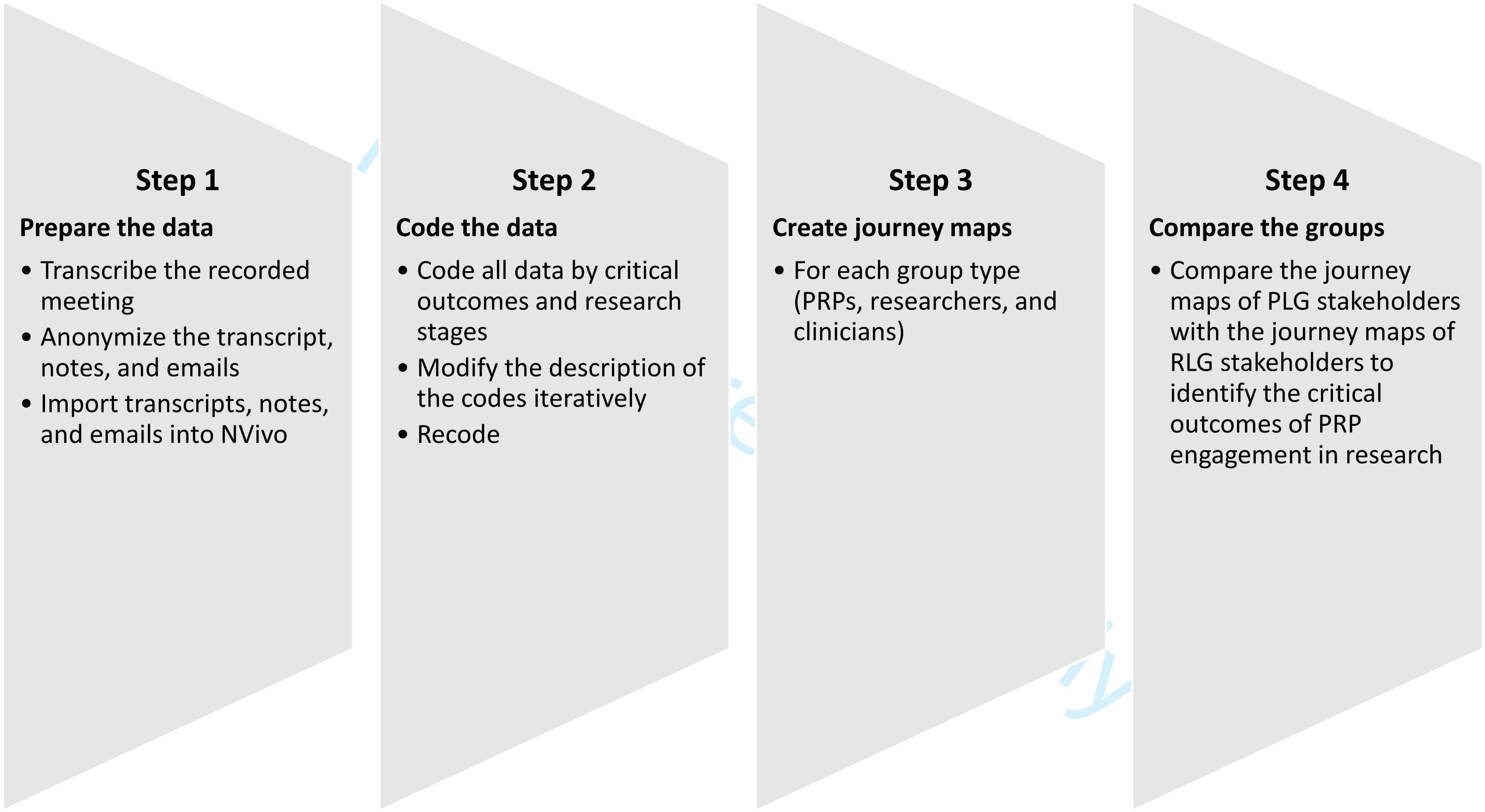

Figure 2. Comparative Journey Maps of PRPs in the PLG and RLG Illustrating Patient-Centeredness

Research Stage	Academic Researcher led Group	Patient Research Partner led Group
Getting to know your team	PRPs introduce themselves to the group.	**Members know each other & their position in the group.**
Deciding on how to work together	One PRP proposes having roles and tasks assigned ahead. The group discusses this strategy but does not formalize roles. Both PRPs **volunteer to tasks** during the different project stages.	PRPs propose additional strategies to communicate and agree with the final plan. Both accept the **roles assigned** to them by the lead.
Helping the study team understand what information is relevant to patients	PRPs share their **lived experiences especially with biologics.** One PRP has side effects and wants to stop taking biologics.	PRPs share their lived experiences. Discussed their experience, **not specifically with the treatment**.
Refining the study question	*PRPs question the definition of tapering and are not comfortable using the word when it was not an option for patients.* **They recommend finalizing the definition of tapering before moving on to next steps.** They look at ways tapering is defined in the literature, discuss, *and agree with the final question, direction and project title.*	*PRPs question what tapering means in the context of the study. They do not like using words such as tapering or withdrawal when discussing tapering.* Both PRPs suggest ways to refine the question and *agree with the final direction and project title.*
Designing the study	PRPs recommend data collection from **both clinicians and patients**, items to be included in the survey. *They recommend items to include in the screening questionnaire and identify questions to ask patients during the interviews.*	PRPs recommend including both UC and Crohn's **patients** in the sample, a ranking exercise after the interviews, an interprovincial lens, conducting interviews over focus groups and **blinding the literature review results from the members collecting data.** *They recommend items to include in the screening questionnaire and identify questions to ask patients during the interviews.*
Developing the study material	**One PRP develops the recruitment flyer, provides questions for both the patient and clinician interview guides, recommends language to be included in the consent, and provides content for the online surveys.** *One PRP develops the interview guides* for clinicians and provides feedback on the patient interview guide. **Both provide feedback on all the study materials.**	*PRPs develop guides* for the focus group and interviews.
Participating in the literature search	PRPs propose questions for the search. **PRPs review papers and extract data.** One PRP identifies papers useful to finalize the definition of tapering and inform the research design.	**PRPs are blinded to the results of the review.**
Training team members on how to recruit and work with patients	**No role.**	**PRPs conduct a mock session of the focus group.**
Finding patients to participate in the study	*PRPs propose platforms and strategies for recruitment.* One PRP was willing to use their connections to identify potential candidates and support recruitment of patients. **One PRP recruits clinician participants.**	*One PRP provides names of potential recruitment platforms.*
Data collection	*One PRP conducts interviews of all the clinicians.*	*PRPs conduct the focus group and interviews.* **They influence the group to drop the "ranking exercise" after the first focus group.**
Analysis and Reviewing results	*One PRP reviews the coded data of one clinician transcript and shares insights with the group.*	PRPs take on an advisory role during data analysis. *They review the analyzed data and agree that it resonates with what they heard during the data collection process.*

Key similarities between the groups are emphasized in orange, italicized text. Key differences are emphasized in blue, bold text.

For peer review only

For peer review only - http://bmjopen.bmj.com/site/about/guidelines.xhtml

Supplementary Table 1

Observation Guide Descriptive field notes for every project group activity

1) Meeting No:

2) Name of the Observer:

3) Group Observing:

4) Date of Observation:

5) Time of Observation: From………. To……………. Total meeting running time:

6) Meeting platform:

7) Number of group members:

8) Names of the group members: (Use ID numbers)

9) Time when each member joins and exists - (capturing how long each stays)

10) Group member roles in each activity during the different research stages and the interaction including the direction of communication, frequency of interaction of each group member, who took the decisions, etc.

- How does the meeting start? What's the mood in the room? Is it all business or do members chat with each other before or after meetings? Does the meeting start on time? Do all members seem to understand the purpose of the meeting, why they are there, and the agenda for the day? Are PRPs consulted when scheduling the activities?
- Does everyone speak or just some group members? Look for familiarity in the conversation for e.g. chatting about previous work together.
- Who is taking all the decisions during the discussion? At what stages/decisions are PRPs being consulted? Did PRPs propose any changes? Capture the changes proposed (changes in study design, methods, materials, etc.) and if not followed, the reasons why if

For peer review only

possible. Do the PRP have any influence on the final decision? Are group members supportive of other opinions in the group? Are there any instances of appreciation of work, giving credit openly during the discussions? Note down examples of members showing respect and supportive of the different viewpoints. Do PRPs initiate and take part in the discussions? How are the non-attendees informed of the decisions? If their input included in the final decision?

- Do PRPs lead any of the discussions? Did you observe silent moments in the conversation? Note: silences do not mean that the group member is uninterested or dis-engaged. It might mean reflective thinking. Jot down your thoughts, and rationale if you make this observation and how the lead/others involve the member and keep the conversation moving.
- Does anybody in the group use terms not understood by the rest of the group? Are there any written materials distributed prior to the meeting for review and questions? If yes, what were these materials? Document any discussions about the written materials for e.g. what does this medical word mean? What is the reading level of these documents?
- What training/support is provided to the PRPs and researchers in the group to help them contribute to the research? Are training requirements discussed? Is there any support request made to the main study team? Is the support provided and how?
- Do any conflicts emerge? What are they? How does the group respond and address the conflict? Note down any challenges that group members faced while engaging on this project.
- What does the morale of the group seem to be at the end of the meetings? Are "next steps" discussed? Are next steps understood? Does anybody on the group linger after the

For peer review only

meeting has closed to talk or do they leave immediately? What kinds of comments are stakeholders making as they leave?

Specific points to capture related to the launch phase (getting to know each other, working together and sharing experiences to help the group understand the supports PRP need)

- What strategy is employed by the group to get to know each? Does any member know another in their group prior to the first meeting? Does the study Lead introduce everyone or do the group members introduce themselves? Or Both?
- Does the group have a formal/informal plan to work together including a communication strategy, roles and responsibilities on the project?? Who developed this plan? Are PRPs involved in its development? If there is no plan, how did the group members operate?
- Did the PRPs in the group share their experience about living with IBD?

Specific points to capture related to the design phase (refining the research question, designing the study, conducting the literature review, developing the study material):

- Do all group members understand the research question, purpose of the study? What changes have been made to the research question? How did the group operationalize the research question? Are PRPs involved in influencing this decision? What is the result of any changes made?
- Who is involved in designing the research? What is each group members contributions to the study design? Are any changes proposed to the study design? Are all suggestions incorporated into the design? Are group members in agreement with the final decision/ validate the changes? Capture the changes made to design based on PRP input and the reason for changes not made. Are group members flexible in making changes keeping the

For peer review only

rigor of the study in mind? Is everyone on board with respect to the final design and approach?

- Is there any mention about the design being straightforward, and easy to accomplish within the time available? Is there any discussion and concern about the study goals and methods? For e.g., need for additional resources like time, expertise etc. to make the study happen. How are these addressed?
- Does the qualitative study design, data collection and analysis accommodate and show respect for participant diversity? Describe the study participants from the conversations.
- Does the group have discussions regarding data/privacy protection?
- What is the role of PRPs in reviewing the literature?
- Who developed the study material? What is the PRP input in their development? Document the materials developed/reviewed by PRP, changes made and reason for those not made. Are the materials written in lay audience language? Evaluate the reading level of the materials.
- Document discussions of personal benefit and benefit to the IBD larger community (for e.g. gained deeper understanding of biologics, gained skills/new knowledge through this engagement, contribute towards the advancement of POR)

Specific points to capture during the Implementation phase (recruitment, data collection, analysis)

- Who is involved in the recruitment of study participants? Who obtained consent/ screened the participants? What is the PRP input during this phase?
- Who is involved in data collection (patients, researchers or a combination of both)?

For peer review only

- Do the recruited study participants represent the living or lived experiences of actual patients, including groups that are typically under-represented in research?
- Who is involved in the analysis of the data? How are PRPs involved in the analysis?
- Is data collection transparent? Does the group use verifiable methods in performing the research and in reporting the results? Does the group pay attention to the ethical rules, regulations, guidelines while conducting the study? (for e.g., informed consent process, patient-friendly materials, data confidentiality and privacy etc.)
- How are the analysis and results shared with the group? Are the results shared with group members in a timely manner? Are the results validated by the PRP? Did they voice any concerns with the results and how were these addressed? Is the study completed within the stipulated time?

Specific points to capture during the Knowledge Translation (KT) Phase (explaining or applying results to real world setting, sharing study findings)

- Are there any discussions or plans about KT? What is the PRP's input during this phase? Are the PRPs in agreement with the plans?

Reflective field notes for every group engagement activity

Document the following elements intended to contextualize what you have observed based on your perspective.

- Note ideas, impressions, thoughts about what you observed. Include insights about why you believe specific phenomenon occurred.
- Include any unanswered questions or concerns that you think are important to record for future observations.

For peer review only

- Record anything that comes to mind that has not been captured elsewhere.

For peer review only

For peer review only

STROBE Statement—Checklist of items that should be included in reports of ***cohort studies***

	Item No	Recommendation	Page No
**Title and abstract**	1	(*a*) Indicate the study's design with a commonly used term in the title or the abstract<br>(*b*) Provide in the abstract an informative and balanced summary of what was done and what was found	1-2
**Introduction**			
Background/rationale	2	Explain the scientific background and rationale for the investigation being reported	3-4
Objectives	3	State specific objectives, including any prespecified hypotheses	3-4
**Methods**			
Study design	4	Present key elements of study design early in the paper	4-5
Setting	5	Describe the setting, locations, and relevant dates, including periods of recruitment, exposure, follow-up, and data collection	5-6
Participants	6	(*a*) Give the eligibility criteria, and the sources and methods of selection of participants. Describe methods of follow-up<br>(*b*) For matched studies, give matching criteria and number of exposed and unexposed	4-5
Variables	7	Clearly define all outcomes, exposures, predictors, potential confounders, and effect modifiers. Give diagnostic criteria, if applicable	6,9
Data sources/ measurement	8*	For each variable of interest, give sources of data and details of methods of assessment (measurement). Describe comparability of assessment methods if there is more than one group	6-9
Bias	9	Describe any efforts to address potential sources of bias	5,8
Study size	10	Explain how the study size was arrived at	4
Quantitative variables	11	Explain how quantitative variables were handled in the analyses. If applicable, describe which groupings were chosen and why	NA
Statistical methods	12	(*a*) Describe all statistical methods, including those used to control for confounding<br>(*b*) Describe any methods used to examine subgroups and interactions<br>(*c*) Explain how missing data were addressed<br>(*d*) If applicable, explain how loss to follow-up was addressed<br>(*e*) Describe any sensitivity analyses	NA
**Results**			
Participants	13*	(a) Report numbers of individuals at each stage of study—eg numbers potentially eligible, examined for eligibility, confirmed eligible, included in the study, completing follow-up, and analysed<br>(b) Give reasons for non-participation at each stage<br>(c) Consider use of a flow diagram	9
Descriptive data	14*	(a) Give characteristics of study participants (eg demographic, clinical, social) and information on exposures and potential confounders<br>(b) Indicate number of participants with missing data for each variable of interest<br>(c) Summarise follow-up time (eg, average and total amount)	9
Outcome data	15*	Report numbers of outcome events or summary measures over time	NA

For peer review only

Main results	16	(*a*) Give unadjusted estimates and, if applicable, confounder-adjusted estimates and their precision (eg, 95% confidence interval). Make clear which confounders were adjusted for and why they were included<br>(*b*) Report category boundaries when continuous variables were categorized<br>(*c*) If relevant, consider translating estimates of relative risk into absolute risk for a meaningful time period	9-20
Other analyses	17	Report other analyses done—eg analyses of subgroups and interactions, and sensitivity analyses	N/A
**Discussion**			
Key results	18	Summarise key results with reference to study objectives	21-23
Limitations	19	Discuss limitations of the study, taking into account sources of potential bias or imprecision. Discuss both direction and magnitude of any potential bias	2,22
Interpretation	20	Give a cautious overall interpretation of results considering objectives, limitations, multiplicity of analyses, results from similar studies, and other relevant evidence	21-22
Generalisability	21	Discuss the generalisability (external validity) of the study results	22
**Other information**			
Funding	22	Give the source of funding and the role of the funders for the present study and, if applicable, for the original study on which the present article is based	24

*Give information separately for exposed and unexposed groups.

Note: An Explanation and Elaboration article discusses each checklist item and gives methodological background and published examples of transparent reporting. The STROBE checklist is best used in conjunction with this article (freely available on the Web sites of PLoS Medicine at http://www.plosmedicine.org/, Annals of Internal Medicine at http://www.annals.org/, and Epidemiology at http://www.epidem.com/). Information on the STROBE Initiative is available at http://www.strobe-statement.org.

For peer review only

For peer review only - http://bmjopen.bmj.com/site/about/guidelines.xhtml
